# Human Neutral Genetic Variation and Forensic STR Data

**DOI:** 10.1371/journal.pone.0049666

**Published:** 2012-11-21

**Authors:** Nuno M. Silva, Luísa Pereira, Estella S. Poloni, Mathias Currat

**Affiliations:** 1 IPATIMUP (Instituto de Patologia e Imunologia Molecular da Universidade do Porto), Universidade do Porto, Porto, Portugal; 2 Faculdade de Medicina, Universidade do Porto, Porto, Portugal; 3 Laboratory of Anthropology, Genetics and Peopling History, Department of Genetics and Evolution - Anthropology Unit, University of Geneva, Geneva, Switzerland; Erasmus University Medical Center, Netherlands

## Abstract

The forensic genetics field is generating extensive population data on polymorphism of short tandem repeats (STR) markers in globally distributed samples. In this study we explored and quantified the informative power of these datasets to address issues related to human evolution and diversity, by using two online resources: an allele frequency dataset representing 141 populations summing up to almost 26 thousand individuals; a genotype dataset consisting of 42 populations and more than 11 thousand individuals. We show that the genetic relationships between populations based on forensic STRs are best explained by geography, as observed when analysing other worldwide datasets generated specifically to study human diversity. However, the global level of genetic differentiation between populations (as measured by a fixation index) is about half the value estimated with those other datasets, which contain a much higher number of markers but much less individuals. We suggest that the main factor explaining this difference is an ascertainment bias in forensics data resulting from the choice of markers for individual identification. We show that this choice results in average low variance of heterozygosity across world regions, and hence in low differentiation among populations. Thus, the forensic genetic markers currently produced for the purpose of individual assignment and identification allow the detection of the patterns of neutral genetic structure that characterize the human population but they do underestimate the levels of this genetic structure compared to the datasets of STRs (or other kinds of markers) generated specifically to study the diversity of human populations.

## Introduction

Short Tandem Repeats (STRs) or microsatellites are popular genetic markers in many applications of genetics, from population characterisation to individual identification and they have also been widely used for gene mapping [1]. The popularity of STRs is due to their hypervariability and ubiquity throughout the genome [2], summing up to 150,000 informative loci with a guaranteed polymorphic level [3]. The variability of these markers is a consequence of a high mutation rate [4], one of the fastest rates among commonly used genetic markers, at least four to six orders of magnitude higher than that of single nucleotide polymorphisms (SNP) [5], [6].

Such features have led to the use of extensive sets of STRs distributed across the genome to characterize patterns of human genetic diversity and population structure, as a means to understand the history of past migrations, the relatedness between populations and associations between genotypes and phenotypes [7], [8], [9], [10]. Despite the good resolution provided by the large amount of markers used, some criticisms have been addressed to these studies that relate to samples sizes, to ascertainment biases, or to a poor representation of the diversity of human populations [11]. Rosenberg et al. [12] tested a series of variables that can affect the clustering level which may be found between populations using the STRUCTURE software [13] in a study of 783 STRs. They found that a low number of loci (10 and 20) reduces the amount of *clusteredeness* among populations, while the opposite effect is obtained by increasing the samples sizes as well as the number of clusters tested. In another study, the type of STRs (number of bases per repeat) was shown to influence the resulting population structure, and the geographic dispersion of the samples was also claimed to be an important factor [14], [15], [16].

The forensic genetics field has generated numerous sample sets typed for a few STRs, distributed over the entire world, in order to assemble a database of genetic profiles ready to be used for individual identification. The number of globally dispersed samples and the high number of individuals screened are interesting aspects of these datasets which may potentially constitute an important source of information about human genetic diversity, despite the relatively low number of markers typed [17], [18], [19], [20], [21], [22]. To ensure the universal utility of the genetic profiles assessed, among the various kits available, two commercial autosomal STR multiplex kits have been extensively used, both comprising a common core of 13 STR loci of the FBI Laboratory’s Combined DNA Index System (CODIS) - CSF1PO, D3S1358, D5S818, D7S820, D8S1179, D13S317, D16S539, D18S51, D21S11, FGA, TH01, TPOX and VWA [17]. Despite some heterogeneity in their evolutionary characteristics (i.e. allele range and number of repeats), all the CODIS STRs have repeat motifs of four nucleotides and at least 16 different alleles observed [19], [20], so as to maximize their power of exclusion. These loci are widely distributed across the human genome, they present independent segregation [20], and are unlikely to have any major functional role, hence escaping natural selection and reflecting mainly the effects of human demographic history [21].

One complex issue distinguishing the population and forensic genetics datasets is the fact that the markers have been chosen differently, namely at random in order to avoid any bias for the former, and for the purpose of individual identification for the latter. These choices may affect the results when both kinds of data compilations are analyzed with identical methods. Moreover, the fields of population and forensic genetics differ basically in two measurable characteristics of their datasets: the number of loci and the sample sizes. Both features can affect population *clusteredeness*, as was shown by Rosenberg et al. [12].

The question of how much information is contained in forensic genetic datasets with respect to issues about human evolution has been debated for some time (e.g. [18], [23]). While some scholars believe that forensic STRs only bear limited information on patterns of genetic diversity, some studies have used these markers for constructing phylogenies (e.g. [18], [24], [25]) or to address specific anthropological questions at local scales (e.g. [26], [27], [28], [29]). A formal evaluation and quantification of this question at a worldwide scale has been postponed due to the difficulties encountered when dealing with the considerable amount of data generated by the forensics field. Recently, two computer tools have facilitated the access to the forensic datasets. One is an online database, strdna-db [30] (available at www.strdna-db.org), that reports STR population data published in the main forensic science journals. As very few of these publications provide individual genotype profiles, the database reports only allelic frequencies and information on geographic location and ethnicity of the samples. Presently, strdna-db sums up a total of 842,826 individuals from 92 countries (2 in Australasia; 1 in North America; 14 in Central and South America; 27 in Europe; 11 in Near East; 6 in North Africa; 11 in sub-Saharan Africa; 7 in South Asia; 5 in East Asia; 8 in Southeast Asia). The second computer tool, PopAffiliator [31] (available at http://cracs.fc.up.pt/popaffiliator), provides 61,212 individual genotype profiles, from more than 40 different studies. This database is still very unbalanced, with a high shift towards Central and South American samples (66% of the database, versus 17% Eurasian; 1.5% sub-Saharan African; 11% East Asian; 2% Near Eastern; 1.5% North African; 1% North American), but it constitutes the most extensive dataset available so far for analysing diversity at the genotype level with the STRs used in forensics.

Thus, despite the low number of loci that are typed, the STR data collected and published by the forensic genetics community cover a considerable amount of globally distributed samples, which suggests that these databases could eventually contain useful information on worldwide patterns of population diversity and may be of interest for making inferences on human evolution. Such a goal calls, beforehand, for a better evaluation and quantification of potential biases introduced in population genetics analyses based on these markers, which have been primarily chosen for other purposes, in particular to meet the forensics interests of individual identification and assignment (therefore leading to ascertainment bias). In this study we present the results of analyses performed to describe the patterns and levels of genetic diversity and structure of human populations inferred from each of the two worldwide forensic datasets described above, i.e. the frequency distributions compiled in strdna-db and the genotype profiles assembled in PopAffiliator. These results are then compared to those obtained with other worldwide non-forensics datasets, in order to highlight possible discrepancies, to quantify them and to determine the likely reasons for these.

## Materials and Methods

### Loci and Samples

The complete datasets provided by strdna-db and PopAffiliator online resources were retrieved and named, respectively, “Frequency” (allele frequencies) and “Genotype” (genotype profiles) datasets. Both datasets were subjected to a phase of maximization of comparable data, leading to the inclusion of only those samples that present information on the 13 CODIS loci commonly tested with the commercial forensic kits. The details and reference of each sample used in this study are given in Tables S1 and S2. The allele nomenclature used refers to the number of repeats. Imperfect alleles, consisting of an increment or a depletion of an incomplete repetitive motif, were also considered. There is some heterogeneity among loci with respect to the complexity of repeat variation. Loci D21S11 and FGA have several imperfect alleles that interrupt the 4 bp repetitive structure. In FGA, these imperfect alleles are distributed at rather low frequencies among samples, whereas they reach substantial frequencies in D21S11. Locus TH01 has instead a single very frequent imperfect allele (allele 9.3, ∼17% and ∼20% for Frequency and Genotype datasets, respectively). For the other 10 loci, the frequency of imperfect alleles is extremely low (<1%).

**Figure 1:**
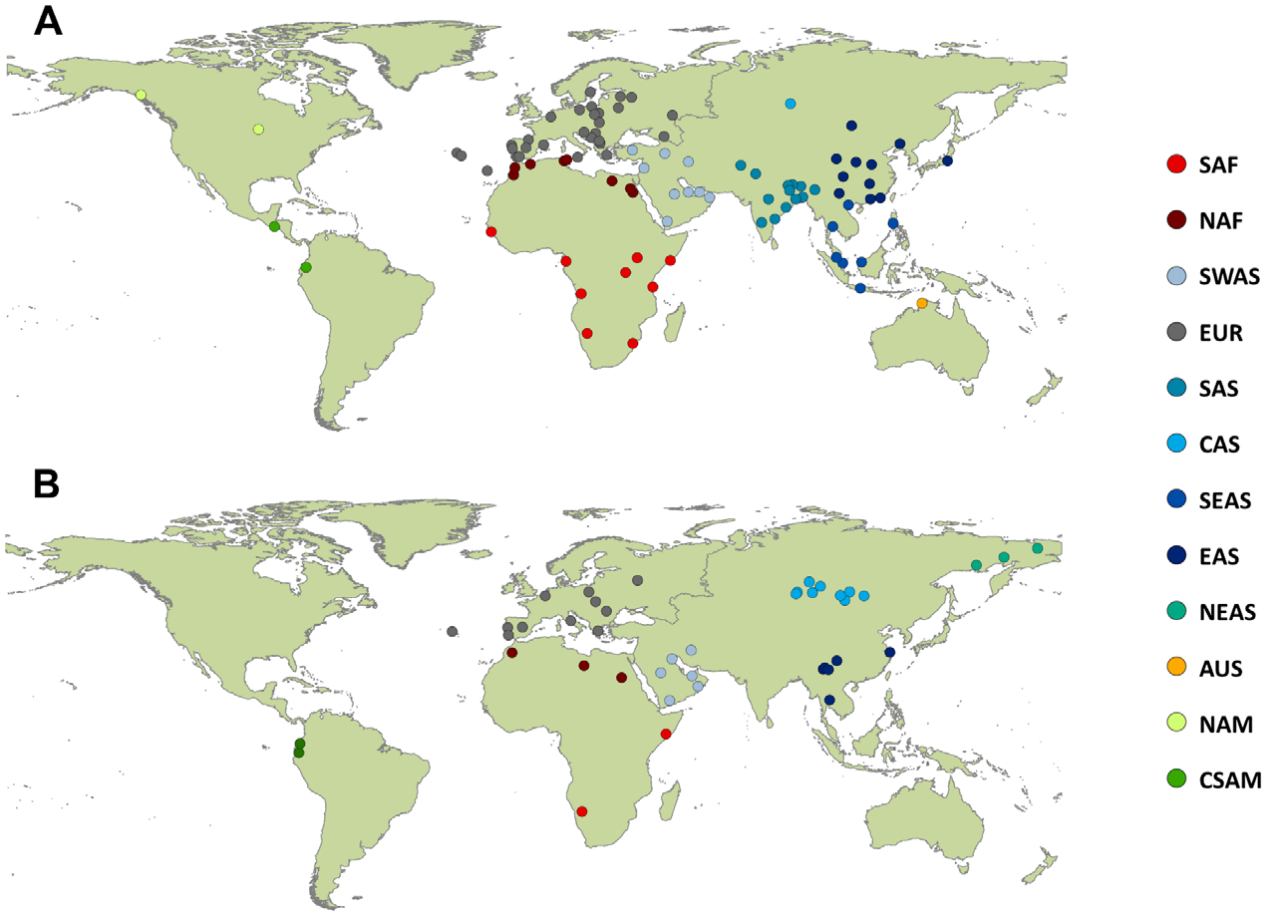
Geographic location of the samples analyzed in this study. 141 samples for the Frequency dataset (A) and 42 samples for the Genotype dataset (B). The populations are assigned to 11 and 8 major geographic groups, respectively.

Further filters were then applied separately to each dataset. For the Frequency dataset, we controlled that the sum of frequencies was equal to 1 for each sample, which led to 190 globally distributed population samples (average over loci of 66,349±1,411 individuals) fitting this requirement (Figure S1A and Table S1). The usefulness of these samples for population genetics studies was further evaluated by identifying those samples supposed to be constituted of individuals of mixed origins or poorly defined provenance (e.g. metropolitan samples or “mestizo” samples from South America) or populations that have recently changed geographic location (e.g. Koreans living in Russia). In this way, 141 samples (summing up to 25,669 individuals) were classified as *well-defined,* being representatives of a given location presumably since a relatively long time, and the other 49 were considered as *possibly admixed* populations, having limited information about geographic/ethnic origin. This led us to consider only the *well-defined* samples (Figure 1A) for the statistical analyses presented in this paper. Note that the representativeness of the various continents is much more balanced when considering the *well-defined* samples only (Africa = 10%, Asia = 42%, Europe = 41%, America = 3%, Oceania = 4%) than in the full database.

We applied similar criteria to the Genotype dataset (described in Figure S1B and Table S2) as those used for the Frequency dataset, which resulted in 42 *well-defined* population samples, summing up 11,132 individuals, comprising almost all the inhabited continents except Australia (Figure 1B). For the Genotype dataset, we also tested Hardy-Weinberg equilibrium in all populations and for all loci using Arlequin 3.5 [32].

As shown in Figure 1, we allocated the population samples to 12 major world geographic regions that correspond to natural geographic subdivisions and spatial extensions of human major language families, following criteria adopted by the immunogenetics community [33], [34]: North Africa (NAF), sub-Saharan Africa (SAF), Europe (EUR), Southwest Asia (SWAS), South Asia (SAS), Central Asia (CAS), Southeast Asia (SEAS), East Asia (EAS), Northeast Asia (NEAS), Australia (AUS), North America (NAM), Central and South America (CSAM). The two datasets are considerably different regarding the number and the distribution of population samples (27 sampling locations are common), with the Frequency dataset representing 11 of the 12 geographic groups (all but NEAS) and the Genotype dataset assigned into 8 groups (all but NAM, AUS, SAS and SEAS).

### Statistical Analyses

#### Genetic diversity within populations and geographic groups

For both datasets, genetic diversity within populations was estimated by two indices: the expected heterozygosity (*He*) [35], computed using Arlequin 3.5 [32], and the variance in the number of repeats (*Vp*), as defined in [36] and computed using a homemade program. Averages over geographic groups were compared by means of Kruskal-Wallis (to test for significant differences among all groups) and Wilcoxon (to test for significant differences between all possible pairs of groups) non-parametric tests, including a Bonferroni correction for multiple testing.

We tested the correlation between population genetic diversity and geographic distance from East Africa (Ethiopia), based on the assumption that this latter region is the most likely place of origin of anatomically modern humans [37]. The geographic distances of all the samples in our datasets to the capital of Ethiopia (Addis Ababa) were calculated as great circle distances between geographic coordinates, using the GeoDist software [38], and following the procedure described in [8], [39] and [40], by considering five obligatory way points used to represent the most likely migration gateways between continental landmasses (in this case, Anadyr in Russia, Cairo in Egypt, Istanbul in Turkey, Phnom Penh in Cambodia, and Prince Rupert in Canada). For example, the distance between a sample in North America and Addis Ababa was computed as the sum of the distances between the North American location and Anadyr, Anadyr and Cairo, and finally Cairo and Addis Ababa. The statistical significance of the resulting correlation coefficients was checked against the critical values of the *t*-test as provided in [41].

#### Genetic differentiation between populations and geographic groups

The genetic relationships between populations were firstly estimated through the computation of matrices of pairwise *R_ST_* indices (distances between alleles were computed as sums of squared differences in repeat numbers), by using the software Arlequin 3.5 [32]. The *R_ST_* values were directly calculated for the Genotype data and their significance tested with the permutation procedure implemented in Arlequin (10,000 iterations). For the Frequency dataset, multi-locus *R_ST_* between each pair of samples was computed using the Michalakis and Excoffier approach [42], as applied in [43]: briefly, since the *R_ST_* index is the ratio of the genetic variance due to differences between populations to the total genetic variance, locus-specific variance components were computed using Arlequin, and then summed over all loci so as to obtain a multi-locus *R_ST_* value. For each locus, *R_ST_* significance was tested through the permutation procedure of Arlequin (10,000 iterations). Population pairwise *R_ST_* values inferred from each of both datasets were then used to calculate coancestry coefficients, (i.e. Reynolds genetic distances [44]), and the resulting matrices of population pairwise genetic distances were submitted to Multidimensional scaling analysis (MDS) using R [45].

In order to explore the relationship of genetic and geographic distances between populations, pairwise great-circle distances between populations locations were calculated with GeoDist in both datasets. Here also, we imposed obligatory waypoints between major landmasses to compute geographic distances between populations from different continents. We used the Mantel test [46] implemented in the GenAlEx 6 software [47] to test the significance of the resulting correlation coefficients between geographic and genetic distances by a permutational resampling process including 1,000 permutations.

The levels of genetic differentiation between all populations and between geographic groups of populations were assessed in both datasets through analyses of molecular variance (AMOVA) [42]. We used a hierarchical framework to obtain the estimations of three fixation indices, reflecting the levels of genetic differentiation, respectively, among populations within geographic groups (*R_SC_*), between geographic groups of populations (*R_CT_*), and globally among all populations (*R_ST_*). The significance of these fixation indices was tested by 10,000 iterations of the permutation procedure implemented in Arlequin. For the Frequency dataset, all the AMOVA computations were performed for each locus independently and, in a similar way as was done for populations pairwise *R_ST_* (see above), the various components of variance were combined across loci to infer multi-locus fixation indices. The statistical significance of the global multi-locus fixation indices were obtained using Fisher’s combined probability test.

The Genotype dataset was also analysed with the STRUCTURE software which infers population clusters that maximize Hardy-Weinberg and linkage equilibrium [13]. For this analysis we used the admixture model and the correlated allele frequency model assuming an ancestral relationship between the populations as was done in [48], and we did not assume *a priori* assignment of individuals to populations. We tested up to nine clusters (*K*) with 10 replicates for each run of 100,000 iterations after a burn-in step of 10,000 iterations. The Evanno approach was applied to determine the number of clusters *K* that best fit the data [49].

#### Comparison with a non-forensics STR dataset

The STR dataset published by Pemberton *et al*. [15] includes information on 627 loci for 1,048 individuals belonging to the 53 worldwide populations of the HGDP-CEPH Human Genome Diversity Cell Line Panel [50]. We extracted the STRs composed of tetra-repeat motifs (i.e. comparable to our forensics STRs) from this HGDP dataset, which total 434 loci. In order to allow comparisons with our forensics results, we calculated averages of the observed number of alleles and expected heterozygosity over geographic groups of populations, as well as the variance of *He* across populations and geographic groups, and performed an AMOVA analysis of these data. Nine geographic groups were defined, still following the criteria adopted by the immunogenetics community [33], [34], so as to match at best our own groups. We then repeated these computations on two subsets of 13 STRs that were chosen for displaying the highest or lowest average *He* over all samples, respectively. These two extreme subsets of markers were taken as representatives of a highly biased choice of markers, either towards high or towards low heterozygosity, and were used in comparisons with our results by means of Wilcoxon and pairwise Levene tests.

## Results

### Genetic Diversity within Populations and Geographic Groups

Tests to detect significant departures from Hardy-Weinberg equilibrium (HWE) were carried out on the Genotype dataset. Among 546 tests, 13% rejected HWE at the 5% level and 5% at the 1% level, both proportions being above the false positive threshold. Except the Chinese sample from Chongming island, for which all the cases were significant even after Bonferroni correction for multiple tests, no specific pattern emerged from this analysis. Indeed, the number of rejection cases was evenly distributed among loci and populations. Moreover, rejections due to excess or deficit in heterozygotes were in similar proportions. When applying a Bonferroni correction with respect to the number of loci tested, cases of HW disequilibrium were still found in 8 (respectively 3) populations out of 42 at the 5% (respectively 1%) level. When applying Bonferroni correction to each locus separately, 3 out of the 13 loci were found in disequilibrium at the 5% level, but none at 1%. Note that two of these loci (D3S1358 and vWA) were in HWE in the study of Sun et al. [51] whereas the third one was not tested by them (TH01). In order for our Genotype dataset to be comparable with other published datasets (see below) for which HWE tests were not performed, we kept all the data for further analyses, including those loci and populations found in disequilibrium.

**Figure 2:**
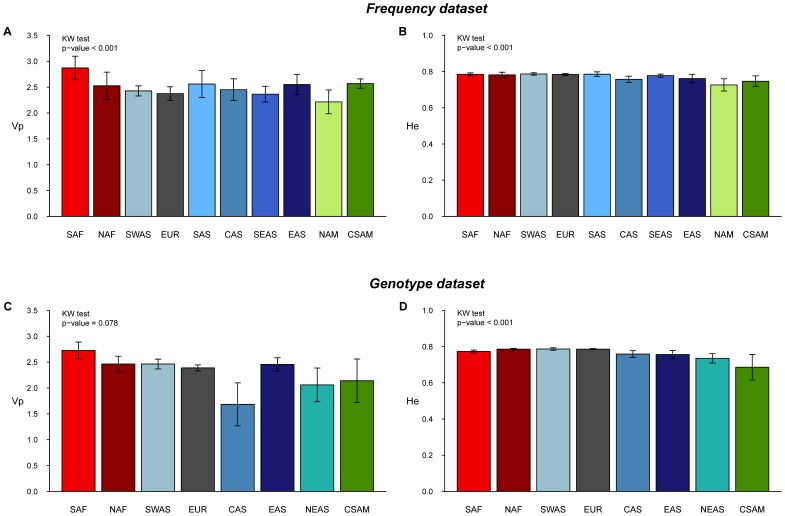
Average genetic diversity (and standard deviation) over populations in geographic groups. 10 groups for the Frequency dataset and 8 for the Genotype dataset (see text). A and C graphs show the distribution of the variance in allele sizes (*Vp*) for the Frequency and Genotype datasets, respectively. B and D graphs show the distribution of the expected heterozygosity (*He*) for the Frequency and Genotype datasets, respectively. *P*-values for the Kruskal-Wallis test (test of significant differences among all groups).

Two measures of intra-population diversity, the variance in allele sizes (i.e., the variance in the number of repeats, *Vp*) and the expected heterozygosity (*He*), were used to investigate the general pattern of genetic diversity across the world for the set of markers analysed. Average values over geographic groups of populations are reported in Figure 2, ordered in each graph, from sub-Saharan Africa to the left, then the Middle-East, Europe, West and East Asia, to the American continent to the right.

The variance in allele sizes (*Vp*) is relatively variable among population groups, especially so for the Frequency dataset (Figure 2A), and the differences for this last dataset are indeed highly significant (Kruskal-Wallis test, *P<0.001*). When groups are compared two by two (Wilcoxon tests, Table S3A), a significant difference in *Vp* is observed for most comparisons involving the South African (SAF) group (except with North Africa (NAF), Australia (AUS), and Central and South America (CSAM)), as well as for the comparison of Europe (EUR) with both South and East Asia (SAS and EAS). For the Genotype dataset (Figure 2C), the apparent differences in *Vp* among groups are not statistically supported (*P = 0.078*), probably due to the effect of a high variance of *Vp* within groups.

Although less variation between groups is apparent on the graphs for the average expected heterozygosity (*He*), the global comparison is significant for both the Frequency and Genotype datasets (Kruskal-Wallis tests, *P<0.001*, Figures 2B and 2D). For the Frequency dataset, *He* shows a decreasing trend from Africa to America, and a rough division can be established between Africa, Southwest Asia and Europe on one side, and the rest of Asia and America on the other, as more significant pairwise differences are seen between groups from these two main areas (Table S3B). For the Genotype dataset, this pattern is not so clear, and indeed only two significantly different pairs of groups (EUR vs. CAS and EUR vs. EAS) are observed in the pairwise comparisons (Table S3D).

**Figure 3:**
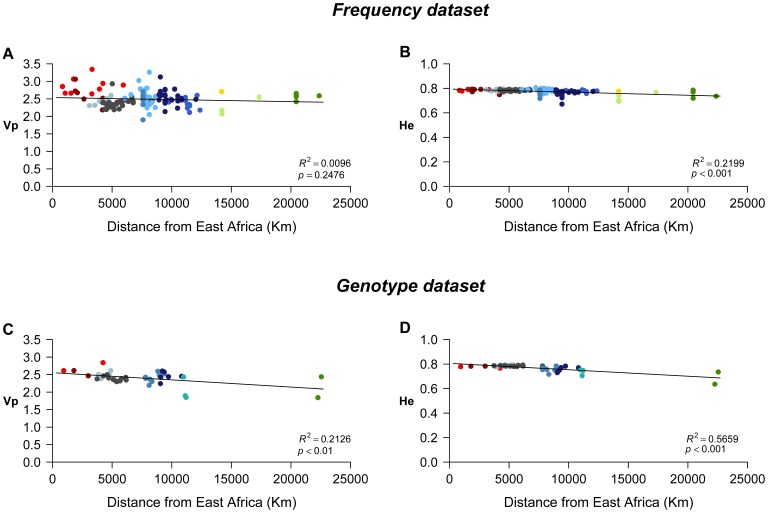
Plots of population diversity against geographic distance to East Africa (Addis Ababa). A: *Vp* against geographic distance for the Frequency dataset; B: *He* against geographic distance for the Frequency dataset; C: *Vp* against geographic distance for the Genotype dataset; D: *He* against geographic distance for the Genotype dataset. The determination coefficient (*R^2^*) estimates the proportion of the variation in genetic diversity that is explained by the variation in geographic distance to East Africa.

The correlation between intra-population diversity and geographic distance from East Africa was found to be significant with both measures *Vp* and *He* (*P*<0.005) in the Genotype dataset, but only with *He* in the Frequency dataset (Figure 3). In both datasets, the correlation is higher with *He* (Frequency dataset: determination coefficient *R^2^* = 0.2199, *P*<0.001; Genotype dataset: *R^2^* = 0.5659, *P*<0.001) than with *Vp* (Genotype dataset: *R^2^* = 0.2126, *P*<0.01). Thus, the distance from East Africa is more influential on the variation of *He* presented by the Genotype dataset.

### Genetic Differentiation between Populations and Geographic Groups

**Figure 4:**
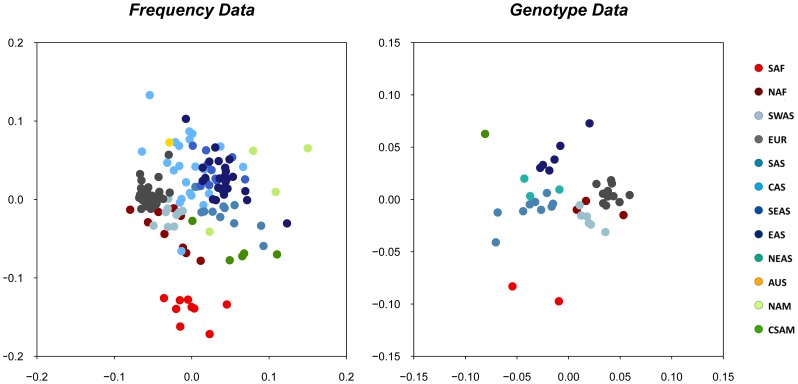
Plots of the multidimesional (MDS) scaling analyses of genetic distances inferred from the forensics datasets. A: MDS of genetic distances computed on the Frequency dataset (stress = 0.18); B: MDS of genetic distances computed on the Genotype dataset (stress = 0.13). Shown in caption: population samples are color-coded following the 12 main geographic groups listed in Figure 1.

Additional tests of inter-population diversity were performed in order to evaluate the genetic differentiation between populations and between geographic groups. Figure 4 displays the resulting plots of multidimensional scaling (MDS) analyses of pairwise Reynolds distances estimated in each of both datasets (the first MDS performed revealed an outlier population sample in each dataset, China Han from Liaoning in the Frequency dataset, and Ecuador Waoranis in the Genotype dataset; the MDS plots shown in Figure 4 were obtained after removal of these samples from the analyses). For both datasets, populations are roughly grouped according to geography, with populations of the same main geographic region tending to locate in the same area of the plot. However, while a distinct cluster made of SAF populations can be observed, the other geographic groups show substantial overlapping, especially in the more numerous Frequency dataset.

The correlation coefficient (*r*) of genetic and geographic distances between populations is of 0.52 in the Frequency dataset, and of 0.64 in the Genotype dataset, and is clearly significant in both cases (*P<0.001*).

AMOVA analyses were performed in order to assess the general levels of population structure and to evaluate population groups defined *a priori* (Table 1). All variance components and associated fixation indices were found statistically significant at the level 5%. The variance component due to differences among groups (3.4% and 3.8% for Frequency and Genotype, respectively) was higher than that due to differences among populations within groups (1.7% and 0.6%). These results indicate that the main geographic groups that were defined are well supported genetically. The overall index of differentiation, *R_ST_*, is of 5.0% and 4.4% for the Frequency and Genotype datasets, respectively.

**Table 1:** Comparison of AMOVA results across studies.

				*Variance Components (%)*		
*Number of Loci*	*Number of Populations*	*RST (%)*	*Number of Groups*	*Among Groups*	*Among populations* *within Groups*	*Reference*
13	141	5.0*	11	3.4*	1.7*	Frequency data
13	43	4.4*	8	3.8*	0.6*	Genotype data
377	52	12.3*	5	9.2*	3.1*	[53]
30	14	15.5^1^	5	10.0^1^	5.5^1^	[54]
60	15	12.1*	3	10.4*	1.7*	[52]
434	53	9.5*	9	6.8*	2.7*	All 434 tetra STRs from [15]
13	53	9.0*	9	6.1*	2.8*	13 STRs with highest *He* from [15]
13	53	13.5*	9	9.5*	4.0*	13 STRs with lowest *He* from [15]

* Values statistically significant at the 5% level.

1 Significance was tested on each locus separately, see the original reference for more details.

We performed the same analyses with two different geographic structures to evaluate their influence on the results. A first run of AMOVA analyses used a structure of 7 geographic groups defined *a priori* following [9], whereas a second run used the 5 geographic groups inferred by the STRUCTURE algorithm in that same study. Fixation indices with 7 and 5 geographic groups are, respectively, very close and only slightly higher than those obtained with our grouping scheme (see Figure S3), thus showing that our results are robust to the group structure chosen *a priori*. More importantly, levels of genetic differentiation among populations in the forensic datasets are systematically about half the values computed for the HGDP dataset, independently of the number of groups considered. Identical results are obtained when the Oceania group of the HGDP and Frequency datasets is excluded from analysis, so as to be fully comparable with the Genotype dataset which does not contain any Oceania sample (Figure S3).

Population structure was also analysed using the Genotype dataset as input for the program STRUCTURE. The results indicate that the best supported structure consists of three ancestry components, present in variable proportions in three groups reflecting roughly Africa, Europe and Asia (Figure S2A). This result actually describes a continuous genetic gradient reflecting geography from Africa to East Asia. The most apparent discontinuity is located in regions where samples are absent in the dataset (Figure S2B) and thus cannot be taken as a true abrupt genetic change between two geographic clusters but rather as a difference between two regions separated by a large unsampled area.

### Comparison with Other Datasets

We compared some aspects of our datasets to the set of 434 tetra STRs analysed in the HGDP samples that were published in Pemberton et al. [15]. Additional studies of worldwide datasets, more heterogeneous in terms of population groups, number of loci were also included as reference [52], [53], [54]. Table 1 shows that the *R_ST_* indices measured in our study (4.4%–5.0%) are between one third and one half the values usually measured with worldwide datasets of STRs (12.1%–15.5%, [52], [53], [54]). The *R_ST_* value for the tetra STRs from the HGDP dataset was of 9.5%, i.e. roughly twice the values estimated on the forensics tetra STRs datasets studied here (Table 1). We also performed the same analysis on two subsets of 13 STRs from the HGDP dataset corresponding to those loci with, respectively, the highest and lowest average value of heterozygosity over populations. Here again, we observed that the *R_ST_* values inferred from our forensics datasets are at least two times lower (Table 1).

**Figure 5:**
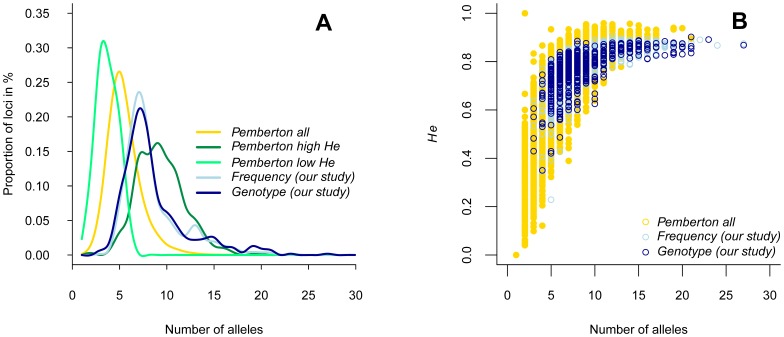
Distributions of the mean number of alleles and with *He* for various datasets. A. Distribution over loci of the mean number of alleles per sample in the two forensics tetra STRs datasets (Frequency and Genotype) and in the HGDP dataset and subsets of tetra STRs published by Pemberton et al. [15] (complete tetra STRs dataset of 434 loci, and subsets of 13 loci biased towards high or low *He*, see text). B. Distribution of *He* over the number of alleles for each locus in each sample.

The comparison with HGDP tetra STRs datasets (i.e. the complete set of 434 tetra STRs and the two subsets of 13 tetra STRs each) shows that the markers used in forensics present a shift towards higher average number of alleles per sample, although this shift fails to reach statistical significance (Wilcoxon tests, Figure 5A and Table S4). However, a significant shift towards higher *He* average per sample is seen in the forensics dataset (Figure 5B and Table S5). Moreover, the variance of *He* between populations is much lower for the markers used in forensics than either for the complete set or for any subset of tetra markers from the HGDP dataset. This difference in variance is statistically significant for all pairwise comparisons with the Frequency dataset, even with the subset of HGDP that is biased towards high *He*. A lower variance is also observed with the Genotype dataset compared to HGDP, but the difference reaches significance only in the comparison with Pemberton’s low *He* subset (Table S6). This lack of statistical significance is probably due to both a reduced number of samples and an overrepresentation of South American samples in the Genotype dataset, which display comparatively lower *He* values (e.g. Ecuador Waoranis *He* = 0.636).

### Model of STR Molecular Evolution

Given the complexity in the repeat structure of some of the 13 CODIS loci analysed in this work, namely FGA, D21S11 and TH01, we repeated all analyses without considering those three loci (10 CODIS loci only). We also computed *F_ST_* indices of genetic differentiation instead of *R_ST_*, being *F_ST_* based on allele frequencies only, whereas *R_ST_* takes into consideration the molecular differences between alleles by assuming a stepwise mutation model of evolution. These additional analyses were performed both with 13 and 10 CODIS markers. They consistently led to similar results, thus showing that our conclusions are robust both to the inclusion of complex loci in the analyses and to the choice of the stepwise model of STR molecular evolution (see Figure S3). When removing FGA, D21S11 and TH01, we obtained very close values of *R_ST_* (5.1% instead of 5.0% with the 13 loci, and 4.2% instead of 4.4%, for the Frequency and Genotype datasets respectively). Globally, *F_ST_* values are lower than *R_ST_* values but display again a similar trend, in that the levels of genetic differentiation estimated with the two forensic datasets (2.7% and 2.3% for the Frequency and Genotype dataset respectively) are a half of those measured in the HGDP dataset (5.3%).

## Discussion

The intensive use of STRs to resolve forensic casework has made the forensic community a main producer of worldwide genetic data. A vast amount of this published data has been recently organized in online databases [30], [31], enabling their use in an automatic and uniform way, less prone to errors. A long debated question in the field is if these markers used by the forensic community for a specific goal, which is to allow individual identification, are also of some value to be used in population genetics studies, as tools to unravel the history and evolution of human populations (e.g. [18], [23]). Indeed, genetic markers used to make inferences on the evolution of our species and to describe its current neutral diversity at the population level are generally chosen in gene-poor regions randomly distributed throughout the genome, in order to avoid ascertainment bias. Previous analyses on some forensic data have shown that these markers allowed detecting a very weak signal of differentiation among European populations [18], [23], but our goal in the present study was to explore and quantify more formally the potential biases introduced by the use of forensics markers instead of randomly chosen markers, at a worldwide scale. Here, we analysed two massive worldwide forensic datasets representing a vast amount of individuals and locations, using indices that account for the molecular (i.e. evolutionary) distance between alleles. This allowed us to address in a more robust way than previous attempts [55] the global patterns of population genetic diversity displayed by the forensic datasets and to examine in details the differences with datasets that have been specifically developed to analyse genetic variation among human populations.

The datasets used in this work were carefully checked regarding two main issues: how well-defined the population samples are in terms of ethnicity and geographic location, and the amount of missing data and loci typed. In order to be considered in population genetics analyses, a sample should be, as much as possible, representative of a geographic region or of a cultural entity (population). Consequently we did not include in the analyses the population samples for which the origin of individuals was either not defined with enough precision or if the sample was mixed. For frequency data (extensively published in forensic journals), the compilation of a dataset of well-defined samples was necessary in order to avoid poorly defined or probably admixed samples which were quite numerous. Regarding the genotype data, most of the samples presented already satisfactory definition but the differential loci typed across profiles and missing data were the main criteria to discard some samples from the analyses.

In agreement with expectations on forensics data (e.g. [51], [55]), we found that the measures of intra-population diversity, expected heterozygosity (*He*) and variance in number of repeats (*Vp*), show relatively low variation between population groups, although significant overall differences are observed. Despite differences between the two measures, both reveal a tendency to decrease from Africa to America. This tendency, consistent with the putative way of migration of modern humans out of Africa [7], [8], [37], [39], was corroborated by significant correlations between diversity and geographic distance from East Africa. When measured using *He*, distance from East Africa explains 57% of the variation in genetic diversity among populations in the Genotype dataset. Even if this determination coefficient is higher than those obtained with the *Vp* measure or with the Frequency dataset, it is still substantially lower than the values obtained in other studies [8], [39], all well above 70%.

In turn, the differences between the two estimators of diversity used here (i.e. *He* and *Vp*) are consistent with a neutral model of human evolution that assumes increased genetic drift with distance from Africa. Indeed, genetic drift leads to reduced heterozygosity, but since it is a stochastic process, the alleles that will drift need not to be the same in different populations. Hence, two populations can end up with similarly low numbers of alleles and heterozygotes (similar *He*), but in one population these alleles could be quite distant in repeat numbers (high *Vp*), whereas in the other population not (low *Vp*). Note that we are describing indices of diversity computed as averages over the 13 CODIS loci while some variance may exist when considering each locus independently.

Our results thus clearly show that geography is the main factor shaping the variation of genetic diversity across populations. In keeping with this observation, a good concordance between geography and genetic distances was shown by the MDS analyses and corroborated by the Mantel tests. Such results are usually found in humans at continental and worldwide scales [56]. Geographic groups were recognizable in the MDS plots (Figure 4) and their consistency is supported by the AMOVA results (Table 1). Overall, however, those groups do not form differentiated clusters, except maybe for the Sub-Saharan African (SAF) group. We nevertheless note that the sharpest differences observed between groups always correspond to geographic areas that have not been sampled (Figure 1). This is particularly clear in the results obtained with the program STRUCTURE, in which the major apparent shift is located between western Eurasia and Eastern Eurasia, at the longitude of India, a region poorly represented in our Genotype dataset.

The proportion of the total genetic variability that is due to differences between populations (*R_ST_*) is similar in both datasets (5% and 4.4%, for Frequency and Genotype, respectively). The datasets differ, however, in the proportion of variation among groups relative to that among populations within groups, which is found higher for the Genotype dataset, probably due to a poorer geographic sampling coverage, particularly so for populations located at the crossroads of continental regions (Figure 1). We found that these results are robust to alternative choices of population groups as well as to the presence of imperfect repeat motives in the data that probably violate the assumption of stepwise evolution of STRs (see Figure S3). Overall our results suggest a smooth gradient of genetic variation between geographic groups rather than abrupt changes.

Our results were compared with those obtained with a dataset made up of genome-wide distributed tetra STRs typed for the populations in the HGDP panel studied by Pemberton *et al.* [15]. The main differences between our two datasets and the HGDP dataset are twofold: *i)* the purpose for which STRs have been designed (individual diversity *versus* population diversity); and *ii)* few loci (13) but many samples and individuals *versus* many loci (434) but less samples and individuals. The *R_ST_* values for the 13 forensics STRs analysed here (in 141 populations from 11 geographic groups for the Frequency dataset, and 43 populations from 8 groups for the Genotype dataset) are about half the values found with the data of [15], as well as those found in other studies [52], [53], [54], all based on a larger number of markers (Table 1). Besides the obvious impact of the number of markers analysed [12], as well as the representativeness of populations, the specific characteristics of the markers can also influence the power to detect population structure. Several non-exclusive factors may potentially account for the low genetic differentiation found in forensics data: *i)* the number and location of samples; *ii)* a high intra-population diversity; *iii)* a low variance in heterozygosity across populations. The first explanation may be discarded as both Frequency and Genotype datasets give *R_ST_* values of the same magnitude (5% and 4.4%) despite a reduced number of samples in the Genotype dataset. We investigated in depth the two other explanations.

The tests performed here on all the tetra STR markers (434 loci) from the worldwide HGDP dataset published in [15] allowed us to address the effect of the characteristics of the markers used in detecting population structure. We investigated the behaviour of the more informative diversity measure, *He*. It is expected that higher diversity within populations is concomitant with reduced magnitude of differentiation among populations, unless the spectrum of extant alleles in diverse populations is substantially different [57], [58]. In general, *He* is slightly higher in our two datasets (0.77) than the value corresponding to the 434 tetra-STRs from the HGDP dataset (0.71), but it is still lower than the extreme value (0.85) obtained with the biased HGDP-extracted subset of 13 loci with the highest *He* (see Table S6). The same trend is observed for the average number of alleles per locus (Figure 5A). The fact that the high *He* subset of 13 loci from HGDP leads to an *R_ST_* value about twice the one measured in our datasets suggests that a high mean heterozygosity within samples could not explain alone the reduced genetic differentiation between samples observed in the forensics data.

However, when considering all 434 tetra STRs in Pemberton’s HGDP dataset the variance of *He* among samples (0.00201) is three to five times higher than those inferred from both the forensics datasets analysed here (0.00043 and 0.00087 for the Frequency and Genotype datasets, respectively). This observation is probably the consequence of an important ascertainment bias in the choice of the 13 CODIS STRs, which have been independently selected in order to be the most discriminating ones. Consequently, this ascertainment bias resulted in a reduced variance between samples compared to STRs randomly chosen in the genome, and thus the genetic differentiation measured by fixation indices is much lower. This fact is strengthened by the results of the comparisons with the two HGDP subsets of 13 loci, picked up to have the highest and the lowest heterozygosities (Table S6 - *He* variance of 0.0023 and 0.0114, respectively), as both have a much higher variance in *He* than those measured in our datasets (and significantly higher than that of the Frequency dataset). This ascertainment bias could also explain the significant number of rejection cases of Hardy-Weinberg equilibrium, which exceed the type-I error threshold. However, to address this hypothesis, the proportion of Hardy-Weinberg disequilibrium should be evaluated in the other published datasets.

### Conclusions

In conclusion, we show that the two forensic datasets investigated contain valuable, albeit limited, information on worldwide genetic diversity, even after a careful selection of well-defined samples as explained in Materials and Methods. Interestingly, they show the same trends than other worldwide neutral datasets: a good correlation between geography and genetics at a worldwide scale and a smooth decreasing gradient of diversity with distance from Africa, along the putative migration routes of modern humans out of Africa. However, those trends are less pronounced in forensic datasets than in other randomly chosen genome-wide datasets. This is a direct consequence of the specificities underlying the choice of STRs for forensic genetics purposes, as those markers have been primarily picked up to maximize individual identification [59], [60]. When these markers are used at the population level, it results in an ascertainment bias towards a low variance in average heterozygosity across populations, contributing to an underestimation of the level of neutral population structure, although the patterns of this structure are conserved. These forensic STRs thus provide results that are consistent with other more extended datasets of markers in the patterns of genetic structure that are inferred, but they are underestimating the levels of genetic variation among human populations.

## Supporting Information

Figure S1 Geographic distribution of the 141 (blue) and 42 (orange) samples of the Frequency and Genotype datasets, respectively. The possibly admixed samples discarded from the starting datasets are also represented in grey. Numbers correspond to the populations’ ID codes listed in Tables S1 and S2, respectively.
(TIF)

Figure S2 Results obtained with STRUCTURE on the Genotype dataset. A: Evanno’s estimation of the number of clusters K that better fits the data, K ranges from 1 to 9. B: Graphical representation of the inferred ancestry of individuals for a K value equal to three.
(TIF)

Figure S3 *R_ST_*/*F_ST_* indices computed with different AMOVA/ANOVA analyses. Many tests were performed with various group structures, considering or not the Oceania group, with or without complex loci and with or without large samples. For the group structures, three definitions were used, either following the immunogenetics community criterion as defined in the main text, or as defined in Rosenberg *et al*’s article (Science 2002), or as inferred in the same study using the program STRUCTURE.
(TIF)

Table S1 Frequency dataset description. The designations and information presented for populations are based on the original publications and the online source of the data (www.strdna-db.org). Geographic coordinates were assigned in this work.
(PDF)

Table S2 Genotype dataset description. The designations and information presented for populations are based on the original publications (http://cracs.fc.up.pt/popaffiliator). Geographic coordinates were assigned in this work.
(PDF)

Table S3 Comparison of average genetic diversity among geographic groups. Pairwise Wilcoxon tests of the difference in average genetic diversity between geographic groups. Tables A and B: average genetic diversity measured by *Vp* and *He* in the Frequency dataset. Tables C and D: average genetic diversity measured by *Vp* and *He* in the Genotype dataset. The p-values below 0.05 are represented in bold and italic.
(PDF)

Table S4 Comparison of number of alleles among datasets. Pairwise Wilcoxon tests of the distributions of the mean number of alleles per sample over loci presented in Figure 5A, with Bonferroni correction. The p-values below 0.05 are represented in bold and italic.
(PDF)

Table S5 Comparison of expected heterozygosity among datasets. Pairwise Wilcoxon tests of the distributions of the expected heterozygosity per sample over loci presented in Figure 5B, with Bonferroni correction. The p-values below 0.05 are represented in bold and italic.
(PDF)

Table S6 Comparison of expected heterozygosity between the two forensic datasets and subsets of HGDP data. A: mean, standard deviation and variance of *He* in both forensic datasets, in the dataset constituted of all Pemberton et al. (2009) tetra loci, and in two subsets of 13 tetra loci of Pemberton et al. (2009) showing highest and lowest average *He* among populations. B: pairwise Levene tests for the variance in *He* between the datasets described in A, corrected for multiple tests. The p-values below 0.05 are represented in bold and italic.
(PDF)
