# Supplementary figures and images for: Human Neutral Genetic Variation and Forensic STR Data
Source: PLoS One. 2012 Nov 21;7(11):e49666. doi: 10.1371/journal.pone.0049666 (PMC3504113; doi:10.1371/journal.pone.0049666)

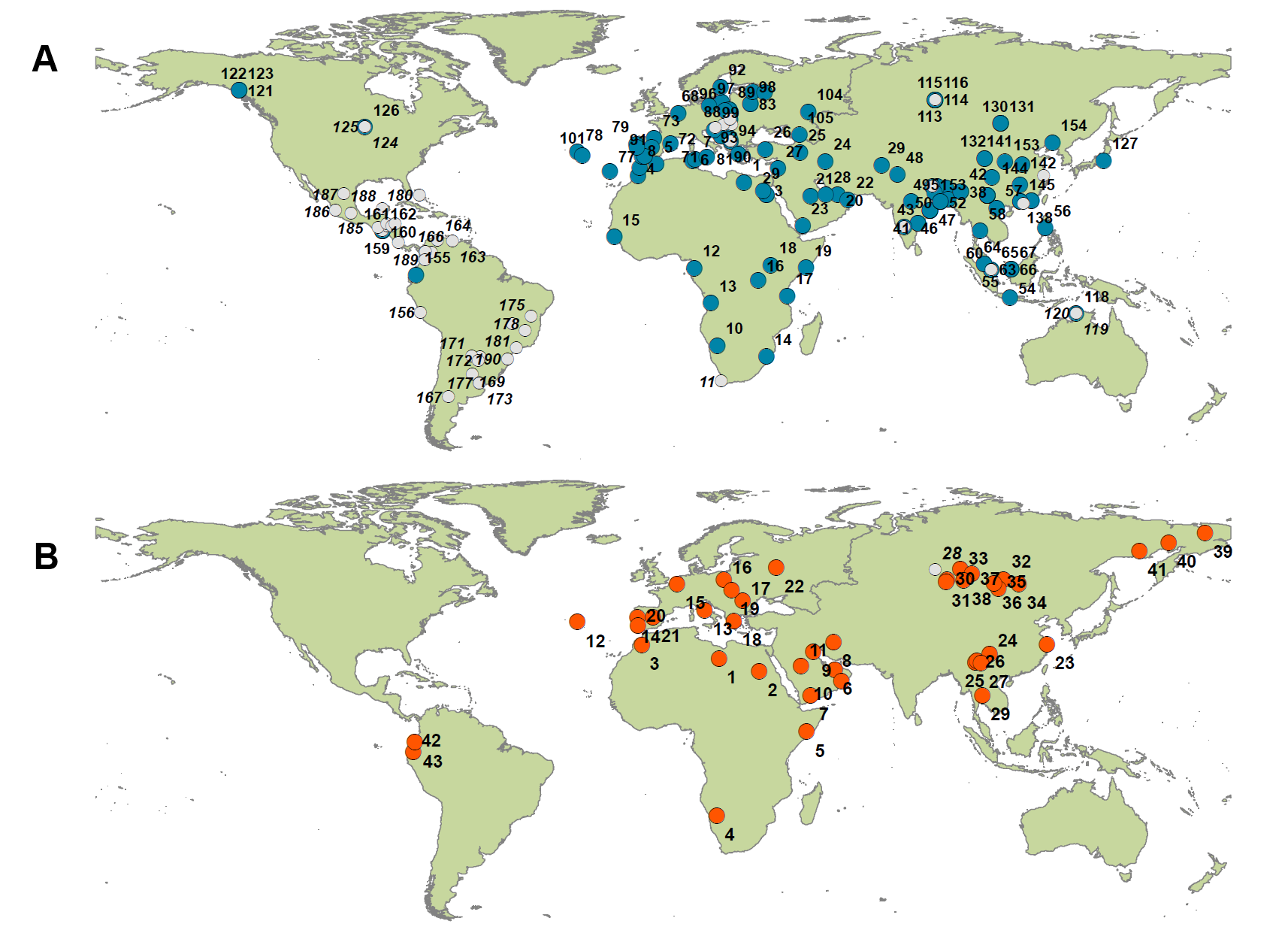

Supplement: Figure S1 — Geographic distribution of the 141 (blue) and 42 (orange) samples of the Frequency and Genotype datasets, respectively. The possibly admixed samples discarded from the starting datasets are also represented in grey. Numbers correspond to the populations’ ID codes listed in Tables S1 and S2, respectively. (TIF) [file pone.0049666.s001.tif]

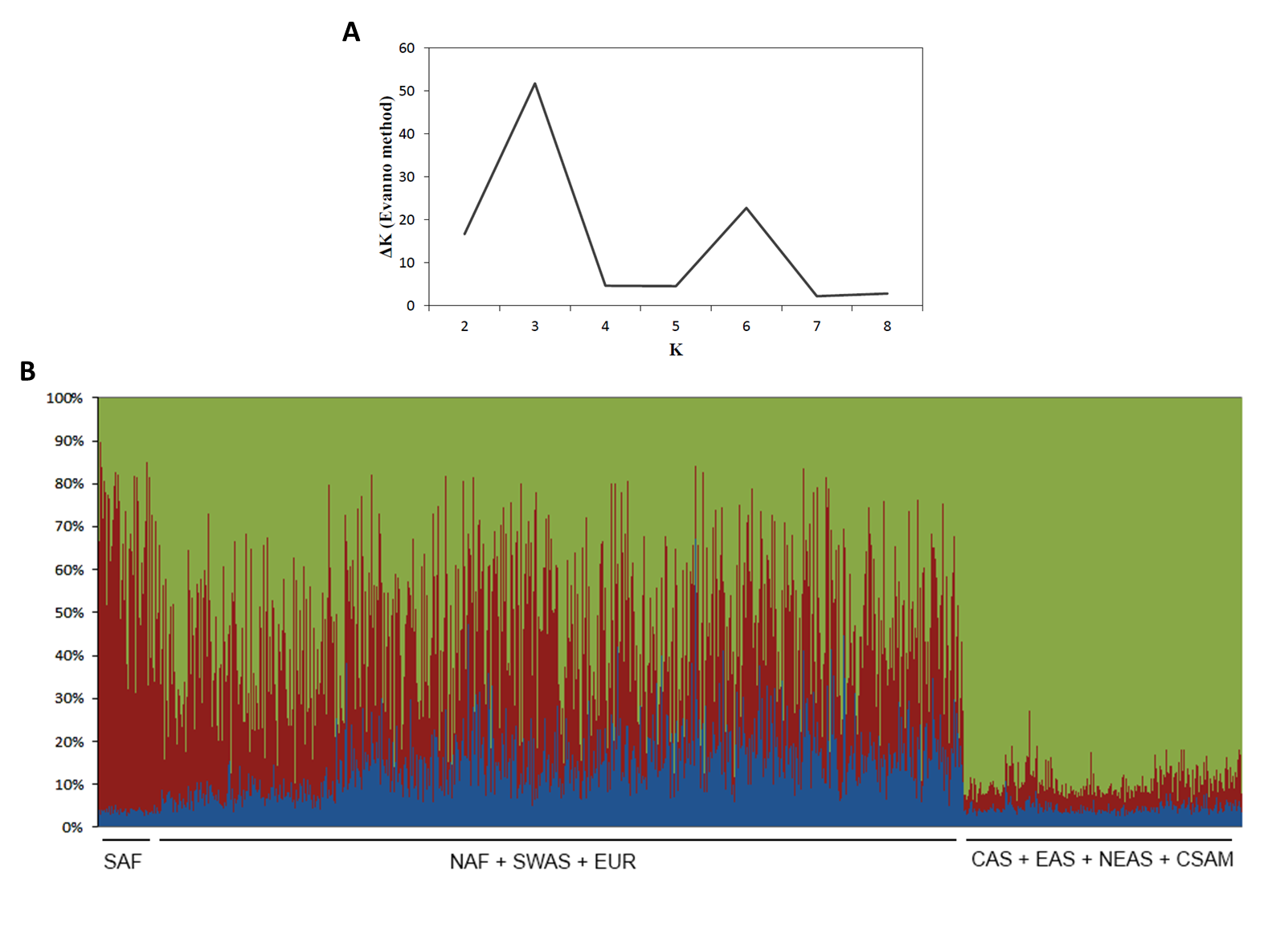

Supplement: Figure S2 — Results obtained with STRUCTURE on the Genotype dataset. A: Evanno’s estimation of the number of clusters K that better fits the data, K ranges from 1 to 9. B: Graphical representation of the inferred ancestry of individuals for a K value equal to three. (TIF) [file pone.0049666.s002.tif]

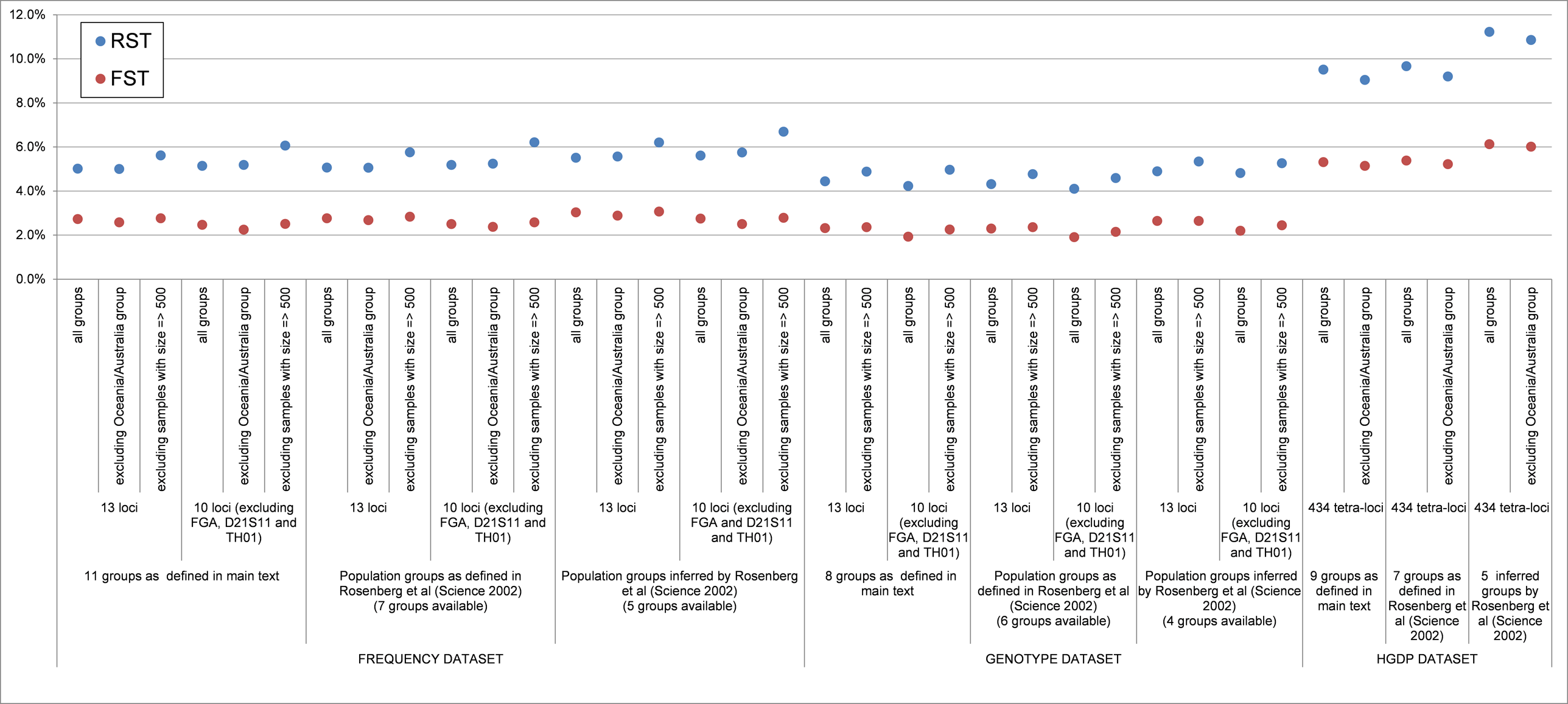

Supplement: Figure S3 — RST/FST indices computed with different AMOVA/ANOVA analyses. Many tests were performed with various group structures, considering or not the Oceania group, with or without complex loci and with or without large samples. For the group structures, three definitions were used, either following the immunogenetics community criterion as defined in the main text, or as defined in Rosenberg et al’s article (Science 2002), or as inferred in the same study using the program STRUCTURE. (TIF) [file pone.0049666.s003.tif]
