# Supplementary material for: Human Neutral Genetic Variation and Forensic STR Data
Source: PLoS One. 2012 Nov 21;7(11):e49666. doi: 10.1371/journal.pone.0049666 (PMC3504113; doi:10.1371/journal.pone.0049666)
Supplement: Table S1 — Frequency dataset description. The designations and information presented for populations are based on the original publications and the online source of the data (www.strdna-db.org). Geographic coordinates were assigned in this work. (PDF) [file pone.0049666.s004.pdf]

Table S1: Frequency dataset description. The designations and information presented for populations are based on the original publications and the online source of the data (www.strdna-db.org). Geographic coordinates were assigned in this work.

Geographic Group	*Population description*[1]	*Sampling location*	*Sample Size*[2]	*Longitude*[3]	*Latitude*[4]	*Reference*	*ID*
North Africa (NAF)	Berber	Siwa	98	25.5	29.2	[1]	1
	Muslim	Adayma	99	32.6	25.2	[1]	2
	Copt	Adayma	100	32.6	25.2	[1]	3
	Berber	Asni	105	-8.0	31.2	[2]	4
	Berber	Sidi Bouhria	104	-2.3	34.9	[2]	5
	Berber	Makthar	42.8 (0.9)	9.2	35.9	[3]	6
	Arab	Zaghouan	44.6 (0.7)	10.1	36.4	[3]	7
	Arab	Casablanca	133.2 (4.7)	-7.6	33.6	[4]	8
	All	Sohag	265	31.7	26.6	[5]	9
Sub-saharan Africa (SAF)	Ovambo - Bantu	Windhoek	195	17.1	-22.6	[6]	10
	Caucasian*	Cape Town	98	18.4	-33.9	[7]	11
	sub-Saharan	Bata	134	9.8	1.9	[8]	12

	sub-Saharan	Cabinda	110	15.0	-9.2	[9]	13
	sub-Saharan	Maputo	142.2 (0.6)	32.6	-26.0	[10]	14
	sub-Saharan	Bissau	100	-15.6	11.9	[11]	15
	Hutu	Kigali	50.9 (1.0)	30.1	-1.9	[12]	16
	All	Dar es Salaam	208.4 (25.8)	39.3	-6.8	[13]	17
	Karimojong - Jie - Dodoth	Kotido	218	34.1	3.0	[14]	18
	sub-Saharan	Mogadishu	404	45.3	2.1	[15]	19
Southwest Asia (SWAS)	Caucasian	Dubai	224	55.3	25.3	[16]	20
	Arab	Riyadh	93.9 (0.3)	46.7	24.7	[16]	21
	Arab	Muscat	79	58.6	23.6	[16]	22
	Arab	Sana'a	101	44.2	15.4	[16]	23
	Arab	Tehran	93	51.4	35.7	[16]	24
	Caucasian	Van	116	43.4	38.5	[17]	25
	Caucasian	Ankara	198	32.4	39.7	[18]	26
	Arab	Damascus	117.2 (3.3)	36.3	33.5	[4]	27
	All	Doha	120	51.5	25.3	[19]	28
South Asia (SAS)	South Asians	Kabul	307.2 (44.1)	69.2	34.5	[20]	29
	South Asians	Thimphu	936	89.7	27.4	[21]	30
	South Asians*	Kathmandu	953	85.3	27.7	[21]	31

Sherpa	Namche Bazaar	105	86.8	27.9	[22]	32
South Asians	Kathmandu	111	85.3	27.7	[22]	33
South Asians	Dhaka	127	90.4	23.7	[23]	34
Gadaba	Orissa	56.0 (1.5)	84.6	20.2	[24]	35
KuviKhond	Orissa	46.2 (5.3)	84.6	20.2	[24]	36
Paroja	Orissa	53.1 (1.3)	84.6	20.2	[25]	37
Lotha - Naga	Nagaland	101.0 (2.4)	94.4	26.2	[24]	38
Baniya	Bihar	90	85.9	25.9	[26]	39
Reddy	Andhra Pradesh	17.9 (3.4)	80.8	16.2	[27]	40
Sakunapakshollu	Andhra Pradesh	21.5 (2.5)	80.8	16.2	[27]	41
Naga	Nagaland	15.2 (2.8)	94.4	26.2	[27]	42
IyengarBrahmin	Karnataka	65	76.3	15.0	[28]	43
Muslim*	Karnataka	45	76.3	15.0	[28]	44
Juangs	Orissa	50	84.6	20.2	[25]	45
Paroja	Orissa	77.6 (0.9)	84.6	20.2	[24]	46
Saora	Orissa	35	84.6	20.2	[25]	47
Kashmiri	Lahore	125	74.3	31.6	[29]	48
Brahmin	Madhya Pradesh	110	78.7	23.0	[30]	49
Rajbanshi	West Bengal	90.7 (0.8)	87.9	23.0	[31]	50

	Dhimal	West Bengal	65.8 (0.6)	87.9	23.0	[32]	51
	Kora	West Bengal	59	87.9	23.0	[32]	52
	Maheli	West Bengal	49	87.9	23.0	[31]	53
Southeast Asia (SEAS)	Southeast Asians	Java	105	109.9	-7.3	[23]	54
	Malay	Kuala Lumpur	210	101.7	3.2	[33]	55
	Southeast Asians	Quezon City	106	121.1	14.7	[34]	56
	Southeast Asians	Hanoi	178	105.8	21.0	[35]	57
	Southeast Asians	Bangkok	210	100.5	13.7	[36]	58
	Indian*	Singapore city	174	103.9	1.3	[37]	59
	Malay	Singapore city	197	103.9	1.3	[38]	60
	Chinese*	Singapore city	209	103.9	1.3	[39]	61
	Chinese*	Singapore city	184	103.9	1.3	[40]	62
	Malay	Singapore city	160.7 (1.1)	103.9	1.3	[40]	63
	Malay	Kuala Lumpur	110	101.7	3.2	[41]	64
	Iban	Petra Jaya	195	110.3	1.6	[42]	65
	Bidayuh	Petra Jaya	195	110.3	1.6	[42]	66
	Melanau	Petra Jaya	128	110.3	1.6	[42]	67
Europe (EUR)	Caucasian	Leuven	222	4.7	50.9	[43]	68
	Romanies*	Debrecen	110	21.6	47.5	[44]	69
	Ashkenazi*	Budapest	178	19.0	47.5	[44]	70

Caucasian	Sicily	220	13.8	37.2	[45]	71
Caucasian	Barcelona	203.9 (0.3)	2.2	41.4	[46]	72
Caucasian	Biscay	71.3 (1.8)	-2.9	43.2	[47]	73
Caucasian	Seville	198.1 (2.5)	-6.0	37.4	[48]	74
Caucasian	Madrid	338.0 (4.1)	-3.7	40.4	[49]	75
Caucasian	Huelva	114	-6.9	37.3	[50]	76
Caucasian	Funchal	125.1 (47.7)	-16.9	32.6	[51]	77
Caucasian	Angra do Heroismo	98.9 (1.0)	-27.2	38.7	[52]	78
Caucasian	Porto	200	-8.6	41.2	[53]	79
Caucasian	Novi Sad	100	19.9	45.3	[54]	80
Caucasian	Skopje	100	21.4	42.0	[55]	81
Romanies*	Skopje	102	21.4	42.0	[55]	82
Caucasian	Minsk	176	27.6	54.0	[56]	83
Caucasian	Sarajevo	183.2 (41.8)	18.4	43.9	[57]	84
Caucasian	Sarajevo	100	18.4	43.9	[58]	85
Romanies*	Cakovec	100	16.4	46.4	[59]	86
Caucasian	Gdansk	145	18.6	54.4	[60]	87
Caucasian	Warsaw	430	21.0	52.2	[61]	88

Caucasian	Warsaw	870	21.0	52.2	[62]	89
Albanians	Pristina	136	21.2	42.7	[63]	90
Caucasian	Seville	47.8 (5.0)	-6.0	37.4	[64]	91
Caucasian	Stockholm	306.8 (6.4)	18.1	59.3	[65]	92
Caucasian	Athens	298.3 (0.5)	23.7	38.0	[66]	93
Caucasian	Presov	137.2 (1.6)	21.2	49.0	[67]	94
Gipsy*	Presov	137.2 (1.6)	21.2	49.0	[67]	95
Caucasian	Lodz	1000	19.5	51.8	[68]	96
Caucasian	Pomorze Zachodnie	600	14.5	53.4	[69]	97
Caucasian	Minsk	1085.5 (38.8)	27.6	54.0	[70]	98
Caucasian	Zagreb	200	16.0	45.8	[71]	99
Caucasian	Coimbra	2113.5 (16.1)	-8.4	40.2	[72]	100
Caucasian	Ponta Delgada	473.9 (1.8)	-25.7	37.7	[72]	101
Caucasian	Pskov	62	28.3	57.8	[73]	102
Caucasian	Velikiy Novgorod	59	32.1	57.6	[73]	103
Caucasian	Saratov	52	46.0	51.5	[73]	104
Caucasian	Vody	60	43.1	44.2	[73]	105

	Altaian	Kemerovo	68	86.1	55.4	[74]	106
	Tofalar	Kemerovo	35	86.1	55.4	[74]	107
	Sojot	Kemerovo	29	86.1	55.4	[74]	108
	Khakas	Kemerovo	50.9 (0.3)	86.1	55.4	[74]	109
	Buryat	Kemerovo	78	86.1	55.4	[74]	110
Central Asia (CAS)	Altaian - Kizhi	Kemerovo	80	86.1	55.4	[74]	111
	Mongol	Kemerovo	39.9 (7.5)	86.1	55.4	[74]	112
	Tuva	Kemerovo	78.8 (4.4)	86.1	55.4	[74]	113
	Chukcha	Kemerovo	15	86.1	55.4	[74]	114
	Even	Kemerovo	14	86.1	55.4	[74]	115
	Khamnigan	Kemerovo	94.9 (0.3)	86.1	55.4	[74]	116
	Koreans*	Kemerovo	49	86.1	55.4	[74]	117
	Aboriginal	Winnellie	1106.0 (159.4)	130.9	-12.4	[75]	118
Australia (AUS)	Asian*	Winnellie	342.9 (167.8)	130.9	-12.4	[75]	119
	East Timorese*	Winnellie	258.0 (34.2)	130.9	-12.4	[75]	120

North America (NAM)	Athabaskan	Juneau	101	-134.6	58.4	[76]	121
	Inupiat	Juneau	109	-134.6	58.4	[76]	122
	Yupik	Juneau	100	-134.6	58.4	[76]	123
	African American*	Minnesota	157	-94.7	46.7	[77]	124
	Hispanic*	Minnesota	151	-94.7	46.7	[77]	125
	Native American	Minnesota	203	-94.7	46.7	[77]	126
East Asia (EAS)	Asian	Saitama	164	139.6	35.9	[78]	127
	Hmar	Ulaanbaatar	80	106.9	47.9	[79]	128
	Mara	Ulaanbaatar	90	106.9	47.9	[79]	129
	Lai	Ulaanbaatar	92	106.9	47.9	[79]	130
	Lusei	Ulaanbaatar	92	106.9	47.9	[79]	131
	Tibetan	Xining	850	101.8	36.6	[80]	132
	Chinese*	Hong Kong Island	325	114.2	22.3	[81]	133
	Chinese	Shantou	286.2 (2.9)	116.7	23.4	[82]	134
	Miao	Guangzhou	140.9 (0.3)	113.3	23.1	[83]	135
	Yi	Guangzhou	164.8 (0.4)	113.3	23.1	[83]	136
	Hui	Guangzhou	143.8 (0.4)	113.3	23.1	[83]	137
	Han	Shantou	121.5 (1.1)	116.7	23.4	[82]	138

	Chinese	Chengdu	200	104.1	30.7	[84]	139
	Chinese*	Suzhou	100	120.6	31.3	[85]	140
	Salar	Qinghai	97.3 (1.9)	101.8	36.6	[86]	141
	Han	Henan	207.9 (0.3)	113.7	34.8	[87]	142
	Yi	Yunnan	120	102.7	25.0	[88]	143
	Tujia	Hunan	98	113.0	28.2	[89]	144
	Han	Guangzhou	501	113.3	23.1	[90]	145
	Han	Yunnan	95.1 (1.0)	102.7	25.0	[91]	146
	Han	Yunnan	113.2 (16.3)	102.7	25.0	[91]	147
	Nu	Yunnan	68.9 (9.9)	102.7	25.0	[91]	148
	Tibetan	Yunnan	55.1 (11.4)	102.7	25.0	[91]	149
	DuLong	Yunnan	41.0 (6.4)	102.7	25.0	[91]	150
	Lisu	Yunnan	49.8 (0.6)	102.7	25.0	[91]	151
	Yi	Yunnan	43.5 (6.3)	102.7	25.0	[91]	152
	Han	Shaanxi	446	108.4	35.6	[92]	153
	Han	Liaoning	358	123.4	41.8	[93]	154
Central and South America (CSAM)	Amerindian Kichwas	Quito	115	-78.5	-0.2	[94]	155
	All*	Lima	100	-77.0	-12.1	[95]	156
	All*	San Jose	496	-84.1	9.9	[96]	157

All*	San Salvador	228	-89.2	13.7	[97]	158
Conchagua	San Salvador	49	-89.2	13.7	[98]	159
Indigenous SanAlejo	San Salvador	52.9 (0.3)	-89.2	13.7	[98]	160
Indigenous Panchimalco	San Salvador	35	-89.2	13.7	[98]	161
Indigenous Izalco	San Salvador	36	-89.2	13.7	[98]	162
All*	Caracas	255	-66.9	10.5	[99]	163
All*	Maracaibo	203	-71.6	10.6	[100]	164
All*	Santander	309.2 (138.3)	-73.5	6.9	[101]	165
All*	Antioquia	364	-75.5	7.2	[102]	166
All*	Neuquen	111	-68.1	-38.9	[103]	167
All*	Buenos Aires	502.5 (26.5)	-58.4	-34.6	[104]	168
All*	Santa Fe	549.2 (30.4)	-60.7	-31.7	[104]	169
All*	Formosa	75.9 (4.7)	-58.2	-26.2	[104]	170
All*	Chaco	55.0 (2.2)	-60.9	-26.0	[104]	171
All*	Corrientes	42.2 (2.2)	-58.8	-27.5	[104]	172
All*	Buenos Aires	143	-58.4	-34.6	[105]	173

All*	Brasilia	13180.8 (3416.8)	-47.9	-15.8	[106]	174
All*	Bahia	150	-42.0	-13.4	[107]	175
Mestizo*	Tegucigalpa	198	-87.5	15.1	[108]	176
All*	Buenos Aires	201.4 (1.0)	-58.4	-34.6	[109]	177
All*	Minas Gerais	14100.4 (6439.8)	-43.8	-17.9	[110]	178
Mestizo*	Guatemala City	200	-90.5	14.6	[111]	179
All*	New Providence	221	-77.4	25.0	[112]	180
Mixed*	Sao Paulo	553.8 (13.4)	-46.6	-23.5	[113]	181
Garifuna*	Bajamar	44.1 (5.3)	-87.8	15.9	[114]	182
Garifuna*	Corozal	52.5 (1.8)	-86.1	15.4	[114]	183
Garifuna*	Iriona	64.2 (0.8)	-85.1	15.9	[114]	184
Mestizo*	Mexico City	376.6 (1.7)	-99.1	19.4	[115]	185
Mestizo*	Jalisco	309	-103.9	20.2	[116]	186
Mestizo*	Puebla	312.9 (0.3)	-101.3	25.4	[116]	187
Mestizo*	Yucatan	262	-89.1	20.7	[116]	188
Mestizo*	Quindio	79.6 (1.1)	-75.7	4.5	[117]	189
All*	Santa Catarina	2992.0 (14.4)	-26.9	-49.4	[118]	190

1: As defined in the original reference (the * marks the *possibly admixed* populations as per our criteria, see main text)
2: Sample size, in number of individuals. Average sample size (standard deviation) is reported when variable among loci
3/4: Geographic coordinates in decimal degrees

References:

1. Coudray C, Guitard E, el-Chennawi F, Larrouy G, Dugoujon JM (2007) Allele frequencies of 15 short tandem repeats (STRs) in three Egyptian populations of different ethnic groups. Forensic Science International 169: 260-265.
2. Coudray C, Guitard E, Keyser-Tracqui C, Melhaoui M, Cherkaoui M, et al. (2007) Population genetic data of 15 tetrameric short tandem repeats (STRs) in Berbers from Morocco. Forensic Science International 167: 81-86.
3. Cherni L, Loueslati Yaacoubi B, Pereira L, Alves C, Khodjet-El-Khil H, et al. (2005) Data for 15 autosomal STR markers (Powerplex 16 System) from two Tunisian populations: Kesra (Berber) and Zriba (Arab). Forensic Science International 147: 101-106.
4. Abdin L, Shimada I, Brinkmann B, Hohoff C (2003) Analysis of 15 short tandem repeats reveals significant differences between the Arabian populations from Morocco and Syria. Leg Med (Tokyo) 5 Suppl 1: S150-155.
5. Omran GA, Rutty GN, Jobling MA (2009) Genetic variation of 15 autosomal STR loci in Upper (Southern) Egyptians. Forensic Sci Int Genet 3: e39-44.
6. Muro T, Fujihara J, Imamura S, Nakamura H, Yasuda T, et al. (2008) Allele frequencies for 15 STR loci in Ovambo population using AmpFlSTR Identifiler Kit. Leg Med (Tokyo) 10: 157-159.
7. Kido A, Dobashi Y, Fujitani N, Hara M, Susukida R, et al. (2007) Population data on the AmpF/STR Identifiler loci in Africans and Europeans from South Africa. Forensic Science International 168: 232-235.
8. Alves C, Gusmao L, Lopez-Parra AM, Soledad Mesa M, Amorim A, et al. (2005) STR allelic frequencies for an African population sample (Equatorial Guinea) using AmpFlSTR Identifiler and Powerplex 16 kits. Forensic Science International 148: 239-242.
9. Beleza S, Alves C, Reis F, Amorim A, Carracedo A, et al. (2004) 17 STR data (AmpF/STR Identifiler and Powerplex 16 System) from Cabinda (Angola). Forensic Science International 141: 193-196.
10. Alves C, Gusmao L, Damasceno A, Soares B, Amorim A (2004) Contribution for an African autosomic STR database (AmpF/STR Identifiler and Powerplex 16 System) and a report on genotypic variations. Forensic Science International 139: 201-205.
11. Goncalves R, Jesus J, Fernandes AT, Brehm A (2002) Genetic profile of a multi-ethnic population from Guine-Bissau (west African coast) using the new PowerPlex 16 System kit. Forensic Science International 129: 78-80.
12. Tofanelli S, Boschi I, Bertoneri S, Coia V, Taglioli L, et al. (2003) Variation at 16 STR loci in Rwandans (Hutu) and implications on profile frequency estimation in Bantu-speakers. Int J Legal Med 117: 121-126.
13. Forward BW, Eastman MW, Nyambo TB, Ballard RE (2008) AMPFlSTR Identifiler STR allele frequencies in Tanzania, Africa. Journal of Forensic Sciences 53: 245.
14. Gomes V, Sanchez-Diz P, Alves C, Gomes I, Amorim A, et al. (2009) Population data defined by 15 autosomal STR loci in Karamoja population (Uganda) using AmpF/STR Identifiler kit. Forensic Sci Int Genet 3: e55-58.
15. Tillmar AO, Backstrom G, Montelius K (2009) Genetic variation of 15 autosomal STR loci in a Somali population. Forensic Sci Int Genet 4: e19-20.
16. Alshamali F, Alkhayat AQ, Budowle B, Watson ND (2005) STR population diversity in nine ethnic populations living in Dubai. Forensic Science International 152: 267-279.
17. Cakir AH, Celebioglu A, Altunbas S, Yardimci E (2003) Allele frequencies for 15 STR loci in Van-Agri districts of the Eastern Anatolia region of Turkey. Forensic Science International 135: 60-63.
18. Akbasak BS, Budowle B, Reeder DJ, Redman J, Kline MC (2001) Turkish population data with the CODIS multiplex short tandem repeat loci. Forensic Science International 123: 227-229.

19. Al-Obaidli A, Sabbagh A, Pravin S, Krishnamoorthy R (2009) Present day inbreeding does not forbid the forensic utility of commonly explored STR loci: a case study of native Qataris. Forensic Sci Int Genet 4: e11-13.
20. Hohoff C, Schurenkamp M, Borchers T, Eppink M, Brinkmann B (2006) Meiosis study in a population sample from Afghanistan: allele frequencies and mutation rates of 16 STR loci. Int J Legal Med 120: 300-302.
21. Kraaijenbrink T, van Driem GL, Opgenort JR, Tuladhar NM, de Knijff P (2007) Allele frequency distribution for 21 autosomal STR loci in Nepal. Forensic Science International 168: 227-231.
22. Ota M, Droma Y, Basnyat B, Katsuyama Y, Asamura H, et al. (2007) Allele frequencies for 15 STR loci in Tibetan populations from Nepal. Forensic Science International 169: 234-238.
23. Dobashi Y, Kido A, Fujitani N, Susukida R, Oya M (2003) Population data of nine STR loci, D3S1358, vWA, FGA, TH01, TPOX, CSF1PO, D5S818, D13S317 and D7S820, in Bangladeshis and Indonesians. Forensic Science International 135: 72-74.
24. Mastana SS, Murry B, Sachdeva MP, Das K, Young D, et al. (2007) Genetic variation of 13 STR loci in the four endogamous tribal populations of Eastern India. Forensic Science International 169: 266-273.
25. Sahoo S, Kashyap VK (2002) Genetic variation at 15 autosomal microsatellite loci in the three highly endogamous tribal populations of Orissa, India. Forensic Science International 130: 189-193.
26. Ashma R, Kashyap VK (2002) Genetic polymorphism at 15 STR loci among three important subpopulation of Bihar, India. Forensic Science International 130: 58-62.
27. Kashyap VK, Sarkar N, Trivedi R (2002) Allele frequencies for STR loci of the Powerplex 16 multiplex system in five endogamous populations of India. Forensic Science International 126: 178-186.
28. Rajkumar R, Kashyap VK (2002) Distribution of alleles of 15 STR loci of the Powerplex 16 Multiplex system in four predominant population groups of South India. Forensic Science International 126: 173-177.
29. Rakha A, Yu B, Hadi S, Li S (2008) Genetic analysis of Kashmiri Muslim population living in Pakistan. Leg Med (Tokyo) 10: 216-219.
30. Dubey B, Meganathan PR, Eaaswarkhanth M, Vasulu TS, Haque I (2009) Forensic STR profile of two endogamous populations of Madhya Pradesh, India. Leg Med (Tokyo) 11: 41-44.
31. Roy S, Eaaswarkhanth M, Dubey B, Haque I (2008) Autosomal STR variations in three endogamous populations of West Bengal, India. Leg Med (Tokyo) 10: 326-332.
32. Singh L, Thangaraj K, Chaubey G, Singh VK, Reddy AG, et al. (2006) Genetic profile of nine autosomal STR loci among Halakki and Kunabhi populations of Karnataka, India. Journal of Forensic Sciences 51: 190-192.
33. Seah LH, Jeevan NH, Othman MI, Jaya P, Ooi YS, et al. (2003) STR Data for the AmpFlSTR Identifiler loci in three ethnic groups (Malay, Chinese, Indian) of the Malaysian population. Forensic Science International 138: 134-137.
34. De Ungria MC, Roby RK, Tabbada KA, Rao-Coticone S, Tan MM, et al. (2005) Allele frequencies of 19 STR loci in a Philippine population generated using AmpFlSTR multiplex and ALF singleplex systems. Forensic Science International 152: 281-284.
35. Shimada I, Brinkmann B, Tuyen NQ, Hohoff C (2002) Allele frequency data for 16 STR loci in the Vietnamese population. Int J Legal Med 116: 246-248.
36. Rerkamnuaychoke B, Rinthachai T, Shotivaranon J, Jomsawat U, Siriboonpiputtana T, et al. (2006) Thai population data on 15 tetrameric STR loci-D8S1179, D21S11, D7S820, CSF1PO, D3S1358, TH01, D13S317, D16S539, D2S1338, D19S433, vWA, TPOX, D18S51, D5S818 and FGA. Forensic Science International 158: 234-237.

37. Lim SE, Tan-Siew WF, Syn CK, Ang HC, Chow ST, et al. (2005) Genetic data for the 13 CODIS STR loci in Singapore Indians. Forensic Science International 148: 65-67.
38. Ang HC, Sornarajah R, Lim SE, Syn CK, Tan-Siew WF, et al. (2005) STR data for the 13 CODIS loci in Singapore Malays. Forensic Science International 148: 243-245.
39. Syn CK, Chuah SY, Ang HC, Lim SE, Tan-Siew WF, et al. (2005) Genetic data for the 13 CODIS STR loci in Singapore Chinese. Forensic Science International 152: 285-288.
40. Yong RY, Aw LT, Yap EP (2004) Allele frequencies of 15 STR loci of three main ethnic populations in Singapore using an in-house marker panel. Forensic Science International 141: 175-183.
41. Maruyama S, Minaguchi K, Takezaki N, Nambiar P (2008) Population data on 15 STR loci using AmpF/STR Identifiler kit in a Malay population living in and around Kuala Lumpur, Malaysia. Leg Med (Tokyo) 10: 160-162.
42. Suadi Z, Siew LC, Tie R, Hui WB, Asam A, et al. (2007) STR data for the AmpFlSTR Identifiler loci from the three main ethnic indigenous population groups (Iban, Bidayuh, and Melanau) in Sarawak, Malaysia. Journal of Forensic Sciences 52: 231-234.
43. Decorte R, Engelen M, Larno L, Nelissen K, Gilissen A, et al. (2004) Belgian population data for 15 STR loci (AmpFlSTR SGM Plus and AmpFlSTR profiler PCR amplification kit). Forensic Science International 139: 211-213.
44. Egyed B, Furedi S, Angyal M, Balogh I, Kalmar L, et al. (2006) Analysis of the population heterogeneity in Hungary using fifteen forensically informative STR markers. Forensic Science International 158: 244-249.
45. Vecchio C, Garofano L, Saravo L, Spitaleri S, Iacovacci G, et al. (2004) Allele frequencies for CODIS loci in a Sicilian population sample. Int Congress Ser: 136-138.
46. Paredes M, Crespillo M, Luque JA, Valverde JL (2003) STR frequencies for the PowerPlex 16 System Kit in a population from Northeast Spain. Forensic Science International 135: 75-78.
47. Perez-Miranda AM, Alfonso-Sanchez MA, Kalantar A, Pena JA, Pancorbo MM, et al. (2005) Allelic frequencies of 13 STR loci in autochthonous Basques from the province of Vizcaya (Spain). Forensic Science International 152: 259-262.
48. Sanz P, Farfan MJ, Prieto V, Torres Y, Lopez-Soto M (2001) STR data for the AmpFlSTR Profiler Plus and COfiler loci from the maghreb (North Africa). Forensic Science International 121: 199-200.
49. Camacho MV, Benito C, Figueiras AM (2007) Allelic frequencies of the 15 STR loci included in the AmpFlSTR Identifiler PCR Amplification Kit in an autochthonous sample from Spain. Forensic Science International 173: 241-245.
50. Coudray C, Calderon R, Guitard E, Ambrosio B, Gonzalez-Martin A, et al. (2007) Allele frequencies of 15 tetrameric short tandem repeats (STRs) in Andalusians from Huelva (Spain). Forensic Science International 168: e21-24.
51. Fernandes AT, Brehm A, Alves C, Gusmao L, Amorim A (2002) Genetic profile of the Madeira Archipelago population using the new PowerPlex16 System kit. Forensic Science International 125: 281-283.
52. Velosa RG, Fernandes AT, Brehm A (2002) Genetic profile of the Acores Archipelago population using the new PowerPlex (R) 16 System Kit. Forensic Science International 129: 68-71.
53. Pinheiro MF, Caine L, Pontes L, Abrantes D, Lima G, et al. (2005) Allele frequencies of sixteen STRs in the population of Northern Portugal. Forensic Science International 148: 221-223.
54. Veselinovic I, Kubat M, Furac I, Skavic J, Martinovic Klaric I, et al. (2004) Allele frequencies of the 15 AmpF lSTR Identifiler loci in the population of Vojvodina Province, Serbia and Montenegro. Int J Legal Med 118: 184-186.

55. Havas D, Jeran N, Efremovska L, Dordevic D, Rudan P (2007) Population genetics of 15 AmpflSTR Identifiler loci in Macedonians and Macedonian Romani (Gypsy). Forensic Science International 173: 220-224.
56. Rebala K, Wysocka J, Kapinska E, Cybulska L, Mikulich AI, et al. (2007) Belarusian population genetic database for 15 autosomal STR loci. Forensic Science International 173: 235-237.
57. Konjhodzic R, Kubat M, Skavic J (2004) Bosnian population data for the 15 STR loci in the Power Plex 16 kit. Int J Legal Med 118: 119-121.
58. Marjanovic D, Bakal N, Pojskic N, Kapur L, Drobnic K, et al. (2006) Allele frequencies for 15 short tandem repeat loci in a representative sample of Bosnians and Herzegovinians. Forensic Science International 156: 79-81.
59. Novokmet N, Pavcec Z (2007) Genetic polymorphisms of 15 AmpFlSTR identifiler loci in Romani population from Northwestern Croatia. Forensic Science International 168: e43-46.
60. Szczerkowska Z, Kapinska E, Wysocka J, Cybulska L (2004) Northern Polish population data and forensic usefulness of 15 autosomal STR loci. Forensic Science International 144: 69-71.
61. Kuzniar P, Ploski R (2004) STR data for the power plex-16 loci in a population from Central Poland. Forensic Science International 139: 261-263.
62. Soltyszewski I, Spolnicka M, Kartasinska E, Konarzewska M, Pepinski W, et al. (2006) Genetic variation of STR loci D3S1358, TH01, D21S11, D18S51, Penta E, D5S818, D13S317, D7S820, D16S539, CSF1PO, Penta D, vWA, D8S1179, TPOX and FGA by GenePrint PowerPlex 16 in a Polish population. Forensic Science International 159: 241-243.
63. Kubat M, Skavic J, Behluli I, Nuraj B, Bekteshi T, et al. (2004) Population genetics of the 15 AmpF/STR identifiler loci in kosovo albanians. International Journal of Legal Medicine 118: 115-118.
64. Perez-Miranda AM, Herrera RJ (2005) Genetic data on 13 STR loci in the Andalusian (South Spain) population. Leg Med (Tokyo) 7: 201-203.
65. Montelius K, Karlsson AO, Holmlund G (2008) STR data for the AmpFlSTR Identifiler loci from Swedish population in comparison to European, as well as with non-European population. Forensic Sci Int Genet 2: e49-52.
66. Sanchez-Diz P, Menounos PG, Carracedo A, Skitsa I (2008) 16 STR data of a Greek population. Forensic Sci Int Genet 2: e71-72.
67. Sotak M, Petrejcikova E, Bernasovska J, Bernasovsky I, Sovicova A, et al. (2008) Genetic variation analysis of 15 autosomal STR loci in Eastern Slovak Caucasian and Romany (Gypsy) population. Forensic Sci Int Genet 3: e21-25.
68. Jacewicz R, Jedrzejczyk M, Ludwikowska M, Berent J (2008) Population database on 15 autosomal STR loci in 1000 unrelated individuals from the Lodz region of Poland. Forensic Sci Int Genet 2: e1-3.
69. Piatek J, Jacewicz R, Ossowski A, Parafiniuk M, Berent J (2008) Population genetics of 15 autosomal STR loci in the population of Pomorze Zachodnie (NW Poland). Forensic Sci Int Genet 2: e41-43.
70. Zhivotovsky LA, Veremeichyk VM, Kuzub NN, Atramentova LA, Udina IG, et al. (2009) A reference data base on STR allele frequencies in the Belarus population developed from paternity cases. Forensic Sci Int Genet 3: e107-109.
71. Haliti N, Carapina M, Masic M, Strinovic D, Klaric IM, et al. (2009) Evaluation of population variation at 17 autosomal STR and 16 Y-STR haplotype loci in Croatians. Forensic Sci Int Genet 3: e137-138.
72. Lopes V, Serra A, Gamero J, Sampaio L, Balsa F, et al. (2009) Allelic frequency distribution of 17 STRs from Identifiler and PowerPlex-16 in Central Portugal area and the Azores archipelago. Forensic Sci Int Genet 4: e1-7.

73. Zhivotovsky LA, Akhmetova VL, Fedorova SA, Zhirkova VV, Khusnutdinova EK (2009) An STR database on the Volga-Ural population. Forensic Sci Int Genet 3: e133-136.
74. Zhivotovsky LA, Malyarchuk BA, Derenko MV, Wozniak M, Grzybowski T (2009) Developing STR databases on structured populations: the native South Siberian population versus the Russian population. Forensic Sci Int Genet 3: e111-116.
75. Eckhoff C, Walsh SJ, Buckleton JS (2007) Population data from sub-populations of the Northern Territory of Australia for 15 autosomal short tandem repeat (STR) loci. Forensic Science International 171: 237-249.
76. Budowle B, Chidambaram A, Strickland L, Beheim CW, Taft GM, et al. (2002) Population studies on three Native Alaska population groups using STR loci. Forensic Science International 129: 51-57.
77. Gross AM, Budowle B (2006) Minnesota population data on 15 STR loci using the Identifiler kit. Journal of Forensic Sciences 51: 1410-1413.
78. Hara M, Yamamoto Y, Takada A, Saito K, Kido A, et al. (2004) Population data for 15 STR loci D3S1358, TH01, D21S11, D18S51, Penta E, D5S818, D13S317, D7S820, D16S539, CSF1PO, Penat D, vWA, D8S1179, TPOX and FGA in Japanese. Progress in Forensic Genetics 10 1261: 204-206.
79. Maity B, Nunga SC, Kashyap VK (2003) Genetic polymorphism revealed by 13 tetrameric and 2 pentameric STR loci in four Mongoloid tribal population. Forensic Science International 132: 216-222.
80. Yan J, Shen C, Li Y, Yu X, Xiong X, et al. (2007) Genetic analysis of 15 STR loci on Chinese Tibetan in Qinghai Province. Forensic Science International 169: e3-6.
81. Chan KM, Chiu CT, Tsui P, Wong DM, Fung WK (2005) Population data for the Identifier 15 STR loci in Hong Kong Chinese. Forensic Science International 152: 307-309.
82. Hu SP, Wu DQ, Xu XH, Liu JW, Li B (2005) Genetic profile of 15 STR loci in the Min Nan population in Southeast China. Forensic Science International 152: 263-265.
83. Liu C, Wang H (2006) STR data for the 15 loci from three minority populations in Guangxi municipality in South China. Forensic Science International 162: 49-52.
84. Zhang H, Li Y, Jiang J, Zhang J, Wu J, et al. (2007) Analysis of 15 STR loci in Chinese population from Sichuan in West China. Forensic Science International 171: 222-225.
85. Gao Y, Zhang Z, Wang Z, Bian S (2005) Genetic data of 15 STR forensic loci in eastern Chinese population. Forensic Science International 154: 78-80.
86. Deng Y, Zhu B, Yu X, Li Y, Fang J, et al. (2007) Genetic polymorphisms of 15 STR loci of Chinese Dongxiang and Salar ethnic minority living in Qinghai Province of China. Leg Med (Tokyo) 9: 38-42.
87. Zeng ZS, Zheng XD, Zhu YL, Wang ZQ, Xiang ZD, et al. (2007) Population genetic data of 15 STR loci in Han population of Henan province (central China). Leg Med (Tokyo) 9: 30-32.
88. Zhu BF, Shen CM, Wu QJ, Deng YJ (2008) Population data of 15 STR loci of Chinese Yi ethnic minority group. Leg Med (Tokyo) 10: 220-224.
89. Wei H, Zhao Q, Li S (2006) Genetic polymorphism for the PowerPlex 16 system from the Chinese Tujia Ethnic Minority Group. Journal of Forensic Sciences 51: 709-710.
90. Liao G, Liu T, Ying B, Sun L, Zou Y, et al. (2008) Population genetic study of 15 STR loci in a Chinese population. Journal of Forensic Sciences 53: 252-253.
91. Kraaijenbrink T, Zuniga S, Su B, Shi H, Xiao CJ, et al. (2008) Allele frequency distribution of 21 forensic autosomal STRs in 7 populations from Yunnan, China. Forensic Sci Int Genet 3: e11-12.
92. Wu YM, Zhang XN, Zhou Y, Chen ZY, Wang XB (2008) Genetic polymorphisms of 15 STR loci in Chinese Han population living in Xi'an city of Shaanxi Province. Forensic Sci Int Genet 2: e15-18.
93. Wang R, Liu L, Zhao J, Peng C (2008) Genetic distribution on 15 STR loci from a population of Southern Liaoning in northeast of China. Forensic Sci Int Genet 2: e25-26.

94. Gonzalez-Andrade F, Sanchez QD, Martinez-Jarreta B (2006) Genetic analysis of the Amerindian Kichwas and Afroamerican descendents populations from Ecuador characterised by 15 STR-PCR polymorphisms. Forensic Science International 160: 231-235.
95. Perez L, Hau J, Izarra F, Ochoa O, Zubiate U, et al. (2003) Allele frequencies for the 13 CODIS STR loci in Peru. Forensic Science International 132: 164-165.
96. Rodriguez A, Arrieta G, Sanou I, Vargas MC, Garcia O, et al. (2007) Population genetic data for 18 STR loci in Costa Rica. Forensic Science International 168: 85-88.
97. Morales JA, Monterrosa JC, Puente J (2004) Population genetic data from El Salvador (Central America) using AmpFlSTR Identifiler PCR Amplification Kit. International Congress Series 1261: 223-225.
98. Lovo-Gomez J, Salas A, Carracedo A (2007) Microsatellite autosomal genotyping data in four indigenous populations from El Salvador. Forensic Science International 170: 86-91.
99. Chiurillo MA, Morales A, Mendes AM, Lander N, Tovar F, et al. (2003) Genetic profiling of a central Venezuelan population using 15 STR markers that may be of forensic importance. Forensic Science International 136: 99-101.
100. Bernal LP, Borjas L, Zabala W, Portillo MG, Fernandez E, et al. (2006) Genetic variation of 15 STR autosomal loci in the Maracaibo population from Venezuela. Forensic Science International 161: 60-63.
101. Vargas CI, Castillo A, Gil AM, Pico AL, García O (2003) Population genetic data for 13 STR loci in a northeast Colombian (department of Santander) population. J Forensic Sci: Int Congress Series 1239: 197-200.
102. Gaviria A, Ibarra AA, Jaramillo N, Palacio OD, Acosta MA, et al. (2004) Nineteen autosomal microsatellite data from Antioquia (Colombia). Forensic Science International 143: 69-71.
103. Toscanini U, Berardi G, Raimondi E (2003) STR data for PowerPlex 16 System from Neuquen population, SW Argentina. Forensic Science International 134: 219-221.
104. Marino M, Sala A, Corach D (2006) Population genetic analysis of 15 autosomal STRs loci in the central region of Argentina. Forensic Science International 161: 72-77.
105. Berardi G, Toscanini U, Raimondi E (2003) STR data for PowerPlex 16 System from Buenos Aires population, Argentina. Forensic Science International 134: 222-224.
106. Whittle MR, Romano NL, Negreiros VA (2004) Updated Brazilian genetic data, together with mutation rates, on 19 STR loci, including D10S1237. Forensic Science International 139: 207-210.
107. Santos MV, Anjos MJ, Andrade L, Vide MC, Corte-Real F, et al. (2004) Population genetic data for the STR loci using the AmpFISTR® identifilerTM kit in Bahia, Brazil. Int Congress Ser 1261: 219-222.
108. Matamoros M, Pinto Y, Inda FJ, Garcia O (2008) Population genetic data for 15 STR loci (Identifiler kit) in Honduras. Leg Med (Tokyo) 10: 281-283.
109. Bozzo WR, Pena MA, Ortiz MI, Lojo MM (2007) Genetic data from Powerplex 16 system and Identifiler kits from Buenos Aires province (Argentina). Leg Med (Tokyo) 9: 151-153.
110. del Castillo DM, Perone C, de Queiroz AR, Mourao PH, de Souza Vasconcellos L, et al. (2009) Populational genetic data for 15 STR markers in the Brazilian population of Minas Gerais. Leg Med (Tokyo) 11: 45-47.
111. Martinez-Espin E, Martinez-Gonzalez LJ, Fernandez-Rosado F, Entrala C, Alvarez JC, et al. (2006) Guatemala mestizo population data on 15 STR loci (Identifiler Kit). Journal of Forensic Sciences 51: 1216-1218.
112. Simms TM, Garcia C, Mirabal S, McCartney Q, Herrera RJ (2008) The genetic legacy of the Transatlantic Slave Trade in the island of New Providence. Forensic Sci Int Genet 2: 310-317.

113. Fridman C, dos Santos PC, Kohler P, Garcia CF, Lopez LF, et al. (2008) Brazilian population profile of 15 STR markers. Forensic Sci Int Genet 2: e1-4.
114. Herrera-Paz EF, Garcia LF, Aragon-Nieto I, Paredes M (2008) Allele frequencies distributions for 13 autosomal STR loci in 3 Black Carib (Garifuna) populations of the Honduran Caribbean coasts. Forensic Sci Int Genet 3: e5-10.
115. Juarez-Cedillo T, Zuniga J, Acuna-Alonzo V, Perez-Hernandez N, Rodriguez-Perez JM, et al. (2008) Genetic admixture and diversity estimations in the Mexican Mestizo population from Mexico City using 15 STR polymorphic markers. Forensic Sci Int Genet 2: e37-39.
116. Rubi-Castellanos R, Anaya-Palafox M, Mena-Rojas E, Bautista-Espana D, Munoz-Valle JF, et al. (2009) Genetic data of 15 autosomal STRs (Identifiler kit) of three Mexican Mestizo population samples from the States of Jalisco (West), Puebla (Center), and Yucatan (Southeast). Forensic Sci Int Genet 3: e71-76.
117. Porras L, Beltran L, Ortiz T, Sanchez-Diz P, Carracedo A, et al. (2008) Genetic polymorphism of 15 STR loci in central western Colombia. Forensic Sci Int Genet 2: e7-8.
118. Ocampos M, Fernandes RC, Latorre AF, da Silva CM, Korndorfer FP, et al. (2009) 15 STR loci frequencies in the population from Santa Catarina, Southern Brazil. Forensic Sci Int Genet 3: e129-131.
