# Supplementary material for: Human Neutral Genetic Variation and Forensic STR Data
Source: PLoS One. 2012 Nov 21;7(11):e49666. doi: 10.1371/journal.pone.0049666 (PMC3504113; doi:10.1371/journal.pone.0049666)
Supplement: Table S2 — Genotype dataset description. The designations and information presented for populations are based on the original publications (http://cracs.fc.up.pt/popaffiliator). Geographic coordinates were assigned in this work. (PDF) [file pone.0049666.s005.pdf]

Table S2: Genotype dataset description. The designations and information presented for populations are based on the original publications (http://cracs.fc.up.pt/popaffiliator). Geographic coordinates were assigned in this work.

Group	***Population description***[1]	***Sample Size***[2]	***Longitude***[3]	***Latitude***[4]	***Reference***	***ID***
North Africa (NAF)	Libya	103	*17.6*	*27.1*	[1]	1
	Egypt Upper	265	*30.4*	*23.1*	[2]	2
	Morroco	209	*-6.9*	*31.5*	[3]	3
Sub-saharan Africa (SAF)	Namibia Bantus	195	*16.9*	*-22.5*	[4]	4
	Somalia	404	*45.5*	*3.9*	[5]	5
Southwest Asia (SWAS)	UAE	217	*54.3*	*23.6*	Unpublished	6
	Yemen	99	*46.7*	*15.3*	Unpublished	7
	Iran	104	*54.0*	*32.3*	Unpublished	8
	Kuwait	502	*47.5*	*29.4*	[6]	9

	Oman	99	*56.4*	*20.0*	Unpublished	10
	Saudi Arabia	106	*43.7*	*24.7*	Unpublished	11
Europe (EUR)	Portugal Azores	475	*-27.3*	*38.7*	[7]	12
	Italy	441	*13.1*	*42.4*	[8]	13
	Portugal Center	2125	*-8.3*	*40.1*	[7]	14
	Belgium Flemish	272	*4.4*	*50.8*	[9]	15
	Poland	1600	*19.3*	*52.1*	[10]	16
	Romania	222	*25.2*	*45.6*	[11]	17
	Greece	300	*22.4*	*39.0*	[12]	18
	Eastern Slovak	138	*21.6*	*48.9*	[13]	19
	Portugal South	48	*-8.1*	*37.5*	Unpublished	20
	Spain	114	*-3.3*	*40.2*	[14]	21
	Russia - European part	384	*35.8*	*56.1*	[15]	22
East Asia (EAS)	China Chongming Island	1000	*121.6*	*31.6*	[16]	23
	China Han	604	*103.5*	*28.7*	[17]	24
	China Lisu	25	*98.9*	*25.9*	[17]	25
	China Nu	36	*99.4*	*26.5*	[17]	26
	China Tibetan	31	*100.6*	*25.7*	[17]	27
	Siberia Korean*	49	*129.1*	*35.8*	[18]	28
	Thailand	210	*101.1*	*15.4*	[19]	29

Central Asia (CAS)	Siberia Altay	68	*90.0*	*52.0*	[18]	30
	Siberia Altay Kizhi	80	*89.6*	*51.5*	[18]	31
	Siberia Buryat	78	*107.9*	*52.2*	[18]	32
	Siberia Khakas	51	*94.2*	*55.5*	[18]	33
	Siberia Khamnigan	95	*112.7*	*50.8*	[18]	34
	Siberia Mongol	42	*106.2*	*49.3*	[18]	35
	Siberia Sojot	29	*104.8*	*50.9*	[18]	36
	Siberia Tofalar	35	*97.9*	*54.0*	[18]	37
	Siberia Tuva	80	*95.2*	*51.9*	[18]	38
Northeast Asia (NEAS)	Siberia Chukchi	15	*171.7*	*66.9*	[18]	39
	Siberia Koryak	32	*160.3*	*64.0*	[18]	40
	Siberia Even	14	*151.0*	*61.3*	[18]	41
Central and South America (CSAM)	Ecuador Kichwas	151	*-79.3*	*-2.5*	Unpublished	42
	Ecuador Waoranis	34	*-78.9*	*0.6*	Unpublished	43

1: As defined in the original reference (the * marks the *possibly admixed* populations as per our criteria, see main text)
2: Number of individuals
3/4: Geographic coordinates in decimal degrees

References:

1. Immel D, Erhuma M, Mustafa T, Kleiber M, Klintschar M (2006) Population genetic analysis in a Libyan population using the PowerPlex 16 system. Int Congress Ser 1288: 421-423
2. Omran GA, Rutty GN, Jobling MA (2009) Genetic variation of 15 autosomal STR loci in Upper (Southern) Egyptians. Forensic Sci Int Genet 3: e39-44.
3. Coudray C, Guitard E, Keyser-Tracqui C, Melhaoui M, Cherkaoui M, et al. (2007) Population genetic data of 15 tetrameric short tandem repeats (STRs) in Berbers from Morocco. Forensic Science International 167: 81-86.
4. Muro T, Fujihara J, Imamura S, Nakamura H, Yasuda T, et al. (2008) Allele frequencies for 15 STR loci in Ovambo population using AmpFlSTR Identifiler Kit. Leg Med (Tokyo) 10: 157-159.
5. Tillmar AO, Backstrom G, Montelius K (2009) Genetic variation of 15 autosomal STR loci in a Somali population. Forensic Sci Int Genet 4: e19-20.
6. Alenizi M, Goodwin W, Ismael S, Hadi S (2008) STR data for the AmpFlSTR Identifiler loci in Kuwaiti population. Leg Med (Tokyo) 10: 321-325.
7. Lopes V, Serra A, Gamero J, Sampaio L, Balsa F, et al. (2009) Allelic frequency distribution of 17 STRs from Identifiler and PowerPlex-16 in Central Portugal area and the Azores archipelago. Forensic Sci Int Genet 4: e1-7.
8. Brisighelli F, Capelli C, Boschi I, Garagnani P, Lareu MV, et al. (2009) Allele frequencies of fifteen STRs in a representative sample of the Italian population. Forensic Sci Int Genet 3: e29-30.
9. Mertens G, Mommers N, Cardoen E, De Bruyn I, Jehaes E, et al. (2006) Flemish population genetic analysis using 15 STRs of the Identifiler® kit. 1288: 328-330.
10. Jacewicz R, Jedrzejczyk M, Ludwikowska M, Berent J (2008) Population database on 15 autosomal STR loci in 1000 unrelated individuals from the Lodz region of Poland. Forensic Sci Int Genet 2: e1-3.
11. Marian C, Anghel A, Bel SM, Ferencz BK, Ursoniu S, et al. (2007) STR data for the 15 AmpFlSTR identifiler loci in the Western Romanian population. Forensic Science International 170: 73-75.
12. Sanchez-Diz P, Acosta MA, Fonseca D, Fernandez M, Gomez Y, et al. (2009) Population data on 15 autosomal STRs in a sample from Colombia. Forensic Sci Int Genet 3: e81-82.
13. Sotak M, Petrejcikova E, Bernasovska J, Bernasovsky I, Sovicova A, et al. (2008) Genetic variation analysis of 15 autosomal STR loci in Eastern Slovak Caucasian and Romany (Gypsy) population. Forensic Sci Int Genet 3: e21-25.
14. Coudray C, Calderon R, Guitard E, Ambrosio B, Gonzalez-Martin A, et al. (2007) Allele frequencies of 15 tetrameric short tandem repeats (STRs) in Andalusians from Huelva (Spain). Forensic Science International 168: e21-24.
15. Zhivotovsky LA, Veremeichyk VM, Kuzub NN, Atramentova LA, Udina IG, et al. (2009) A reference data base on STR allele frequencies in the Belarus population developed from paternity cases. Forensic Sci Int Genet 3: e107-109.
16. Li C, Li L, Zhao Z, Lin Y, Que T, et al. (2009) Genetic polymorphism of 17 STR loci for forensic use in Chinese population from Shanghai in East China. Forensic Sci Int Genet 3: e117-118.
17. Kraaijenbrink T, Zuniga S, Su B, Shi H, Xiao CJ, et al. (2008) Allele frequency distribution of 21 forensic autosomal STRs in 7 populations from Yunnan, China. Forensic Sci Int Genet 3: e11-12.
18. Zhivotovsky LA, Malyarchuk BA, Derenko MV, Wozniak M, Grzybowski T (2009) Developing STR databases on structured populations: the native South Siberian population versus the Russian population. Forensic Sci Int Genet 3: e111-116.
19. Rerkamnuaychoke B, Rinthachai T, Shotivaranon J, Jomsawat U, Siriboonpiputtana T, et al. (2006) Thai population data on 15 tetrameric STR loci-D8S1179, D21S11, D7S820,

CSF1PO, D3S1358, TH01, D13S317, D16S539, D2S1338, D19S433, vWA, TPOX, D18S51, D5S818 and FGA. Forensic Science International 158: 234-237.
