# Supplementary material for: Human Neutral Genetic Variation and Forensic STR Data
Source: PLoS One. 2012 Nov 21;7(11):e49666. doi: 10.1371/journal.pone.0049666 (PMC3504113; doi:10.1371/journal.pone.0049666)
Supplement: Table S3 — Comparison of average genetic diversity among geographic groups. Pairwise Wilcoxon tests of the difference in average genetic diversity between geographic groups. Tables A and B: average genetic diversity measured by Vp and He in the Frequency dataset. Tables C and D: average genetic diversity measured by Vp and He in the Genotype dataset. The p-values below 0.05 are represented in bold and italic. (PDF) [file pone.0049666.s006.pdf]

Table S3: **Comparison of average genetic diversity among geographic groups.** Pairwise Wilcoxon tests of the difference in average genetic diversity between geographic groups. Tables A and B: average genetic diversity measured by *Vp* and *He* in the Frequency dataset. Tables C and D: average genetic diversity measured by *Vp* and *He* in the Genotype dataset. The p-values below 0.05 are represented in bold and italic.

A

	SAF	***NAF***	***SWAS***	***EUR***	***SAS***	***CAS***	***SEAS***	***EAS***	***AUS***	***NAM***	***CSAM***
SAF	-	-	-	-	-	-	-	-	-	-	-
NAF	0.78054	-	-	-	-	-	-	-	-	-	-
SWAS	***0.00226***	1	-	-	-	-	-	-	-	-	-
EUR	***7.40E-06***	1	1	-	-	-	-	-	-	-	-
SAS	***0.04877***	1	1	***0.02834***	-	-	-	-	-	-	-
CAS	***0.00458***	1	1	0.61179	1	-	-	-	-	-	-
SEAS	***0.00065***	1	1	1	0.82567	1	-	-	-	-	-
EAS	***0.00702***	1	1	***0.00016***	1	1	0.29948	-	-	-	-
AUS	1	1	1	1	1	1	1	1	-	-	-
NAM	0.15385	1	1	1	1	1	1	1	1	-	-
CSAM	0.10989	1	1	0.05566	1	1	0.73031	1	1	1	-

B

	SAF	***NAF***	***SWAS***	***EUR***	***SAS***	***CAS***	***SEAS***	***EAS***	***AUS***	***NAM***	***CSAM***
SAF	-	-	-	-	-	-	-	-	-	-	-
NAF	1	-	-	-	-	-	-	-	-	-	-
SWAS	1	1	-	-	-	-	-	-	-	-	-
EUR	1	1	1	-	-	-	-	-	-	-	-
SAS	1	1	1	1	-	-	-	-	-	-	-
CAS	***0.01244***	0.1729	***0.00786***	***5.90E-05***	***0.00124***	-	-	-	-	-	-
SEAS	1	1	1	1	1	0.07703	-	-	-	-	-
EAS	***0.00281***	***0.04789***	***0.0001***	***2.10E-09***	***0.00019***	1	0.07531	-	-	-	-
AUS	1	1	1	1	1	1	1	1	-	-	-
NAM	0.15385	0.30769	0.15385	0.00167	***0.04387***	1	0.16117	1	1	-	-
CSAM	0.65934	1	0.65934	0.48761	0.28429	1	1	1	1	1	-

C

	SAF	*NAF*	*SWAS*	*EUR*	*CAS*	*EAS*	*NEAS*	*CSAM*
SAF	-	-	-	-	-	-	-	-
NAF	1	-	-	-	-	-	-	-
SWAS	1	1	-	-	-	-	-	-
EUR	0.72	1	1	-	-	-	-	-
CAS	1	1	1	1	-	-	-	-
EAS	1	1	1	1	1	-	-	-
NEAS	1	1	1	1	1	1	-	-
CSAM	1	1	1	1	1	1	1	-

D

	SAF	*NAF*	*SWAS*	*EUR*	*CAS*	*EAS*	*NEAS*	*CSAM*
SAF	-	-	-	-	-	-	-	-
NAF	1	-	-	-	-	-	-	-
SWAS	1	1	-	-	-	-	-	-
EUR	0.7179	1	1	-	-	-	-	-
CAS	1	1	0.0783	***0.0063***	-	-	-	-
EAS	1	0.9333	0.2284	***0.007***	1	-	-	-
NEAS	1	1	0.6667	0.1538	1	1	-	-
CSAM	1	1	1	0.7179	1	1	1	-
