# Supplementary material for: Human Neutral Genetic Variation and Forensic STR Data
Source: PLoS One. 2012 Nov 21;7(11):e49666. doi: 10.1371/journal.pone.0049666 (PMC3504113; doi:10.1371/journal.pone.0049666)
Supplement: Table S4 — Comparison of number of alleles among datasets. Pairwise Wilcoxon tests of the distributions of the mean number of alleles per sample over loci presented in Figure 5A, with Bonferroni correction. The p-values below 0.05 are represented in bold and italic. (PDF) [file pone.0049666.s007.pdf]

Table S4: Comparison of number of alleles among datasets. Pairwise Wilcoxon tests of the distributions of the mean number of alleles per sample over loci presented in Figure 5A, with Bonferroni correction. The p-values below 0.05 are represented in bold and italic.

	Pemberton all	***Pemberton high He***	***Pemberton low He***	***Frequency (our study)***	***Genotype (our study)***
Pemberton all	-				
Pemberton high He	1	-			
Pemberton low He	0.135	0.376	-		
Frequency (our study)	1	1	***0.019***	-	
Genotype (our study)	1	1	***0.052***	1	-
