# Supplementary material for: Human Neutral Genetic Variation and Forensic STR Data
Source: PLoS One. 2012 Nov 21;7(11):e49666. doi: 10.1371/journal.pone.0049666 (PMC3504113; doi:10.1371/journal.pone.0049666)
Supplement: Table S5 — Comparison of expected heterozygosity among datasets. Pairwise Wilcoxon tests of the distributions of the expected heterozygosity per sample over loci presented in Figure 5B, with Bonferroni correction. The p-values below 0.05 are represented in bold and italic. (PDF) [file pone.0049666.s008.pdf]

Table S5: Comparison of expected heterozygosity among datasets. Pairwise Wilcoxon tests of the distributions of the expected heterozygosity per sample over loci presented in Figure 5B, with Bonferroni correction. The p-values below 0.05 are represented in bold and italic.

	Pemberton all	***Pemberton high He***	***Pemberton low He***	***Frequency (our study)***	***Genotype (our study)***
Pemberton all	-				
Pemberton high He	***5.50E-06***	-			
Pemberton low He	1	***0.00048***	-		
Frequency (our study)	***2.60E-04***	1	***0.01967***	-	
Genotype (our study)	***0.00026***	1	***0.01692***	1	-
