# Supplementary material for: Human Neutral Genetic Variation and Forensic STR Data
Source: PLoS One. 2012 Nov 21;7(11):e49666. doi: 10.1371/journal.pone.0049666 (PMC3504113; doi:10.1371/journal.pone.0049666)
Supplement: Table S6 — Comparison of expected heterozygosity between the two forensic datasets and subsets of HGDP data. A: mean, standard deviation and variance of He in both forensic datasets, in the dataset constituted of all Pemberton et al. (2009) tetra loci, and in two subsets of 13 tetra loci of Pemberton et al. (2009) showing highest and lowest average He among populations. B: pairwise Levene tests for the variance in He between the datasets described in A, corrected for multiple tests. The p-values below 0.05 are represented in bold and italic. (PDF) [file pone.0049666.s009.pdf]

Table S6: Comparison of expected heterozygosity between the two forensic datasets and subsets of HGDP data. A: mean, standard deviation and variance of *He* in both forensic datasets, in the dataset constituted of all Pemberton et al. (2009) tetra loci, and in two subsets of 13 tetra loci of Pemberton et al. (2009) showing highest and lowest average *He* among populations. B: pairwise Levene tests for the variance in *He* between the datasets described in A, corrected for multiple tests. The p-values below 0.05 are represented in bold and italic.

A

	Pemberton all	***Pemberton high He***	***Pemberton low He***	***Frequency (our study)***	***Genotype (our study)***
Mean	0.7107	0.8527	0.4973	0.7746	0.7670
std	0.0453	0.0659	0.1647	0.0207	0.0299
variance	0.0021	0.0023	0.0114	0.0004	0.0009

B

	Pemberton all	***Pemberton high He***	***Pemberton low He***	***Frequency (our study)***	***Genotype (our study)***
Pemberton all	-				
Pemberton high He	0.8300	-			
Pemberton low He	***< 2.2e-16***	***< 2.2e-16***	-		
Frequency (our study)	***0.0020***	***0.0120***	***< 2.2e-16***	-	
Genotype (our study)	0.3180	0.5160	***< 2.2e-16***	0.1030	-
